# Functional Characterization of ABCC Proteins from *Trypanosoma cruzi* and Their Involvement with Thiol Transport

**DOI:** 10.3389/fmicb.2018.00205

**Published:** 2018-02-14

**Authors:** Kelli Monteiro da Costa, Raphael C. Valente, Eduardo J. Salustiano, Luciana B. Gentile, Leonardo Freire-de-Lima, Lucia Mendonça-Previato, José O. Previato

**Affiliations:** ^1^Laboratório de Glicobiologia, Instituto de Biofísica Carlos Chagas Filho, Universidade Federal do Rio de Janeiro, Rio de Janeiro, Brazil; ^2^Faculdade de Ciências Médicas, Universidade do Estado do Rio de Janeiro, Rio de Janeiro, Brazil

**Keywords:** ABC transporters, *Trypanosoma cruzi*, multidrug resistance phenotype, multidrug resistance protein, P-glycoprotein, thiol transporter, Chagas disease

## Abstract

Chagas disease is a neglected disease caused by the protozoan *Trypanosoma cruzi* and affects 8 million people worldwide. The main chemotherapy is based on benznidazole. The efficacy in the treatment depends on factors such as the parasite strain, which may present different sensitivity to treatment. In this context, the expression of ABC transporters has been related to chemotherapy failure. ABC transporters share a well-conserved ABC domain, responsible for ATP binding and hydrolysis, whose the energy released is coupled to transport of molecules through membranes. The most known ABC transporters are ABCB1 and ABCC1, involved in the multidrug resistance phenotype in cancer, given their participation in cellular detoxification. In *T. cruzi*, 27 ABC genes were identified in the genome. Nonetheless, only four ABC genes were characterized: ABCA3, involved in vesicular trafficking; ABCG1, overexpressed in strains naturally resistant to benznidazole, and P-glycoprotein 1 and 2, whose participation in drug resistance is controversial. Considering P-glycoprotein genes are related to ABCC subfamily in *T. cruzi* according to the demonstration using BLASTP alignment, we evaluated both ABCB1-like and ABCC-like activities in epimastigote and trypomastigote forms of the Y strain. The transport activities were evaluated by the efflux of the fluorescent dyes Rhodamine 123 and Carboxyfluorescein in a flow cytometer. Results indicated that there was no ABCB1-like activity in both *T. cruzi* forms. Conversely, results demonstrated ABCC-like activity in both epimastigote and trypomastigote forms of *T. cruzi*. This activity was inhibited by ABCC transport modulators (probenecid, indomethacin, and MK-571), by ATP-depleting agents (sodium azide and iodoacetic acid) and by the thiol-depleting agent N-ethylmaleimide. Additionally, the presence of ABCC-like activity was supported by direct inhibition of the thiol-conjugated compound efflux with indomethacin, characteristic of ABCC subfamily members. Taken together, the results provide the first description of native ABCC-like activity in *T. cruzi* epimastigote and trypomastigote forms, indicating that the study of the biological role for that thiol transporter is crucial to reveal new molecular mechanisms for therapeutic approaches in the Chagas disease.

## Introduction

Chagas disease or American trypanosomiasis is a zoonosis caused by the protozoan *Trypanosoma cruzi*, discovered by Dr. Carlos Chagas in 1909 (Chagas, 1909). Although parasite transmission may occur through diverse pathways, the most common are vectorial, oral and congenital transmissions (Coura, 2007). The WHO estimates that eight million people are chronically infected with *T. cruzi* in the world, mostly in Latin America, where the disease is endemic (WHO, 2012). Notwithstanding, several cases have been reported in non-endemic areas such as European countries, North America, Japan and Australia as result of migratory processes (Bern and Montgomery, 2009).

Chagas disease occurs in two phases: acute and chronic. Acute Chagas disease is characterized by high parasitemia, being recognized in few patients due to absence or non-specificity of clinical symptoms (Bern, 2015). Chronic Chagas disease begins about 2 or 3 months after infection and has been divided in the determinate and indeterminate forms. About 70% of patients present positive serology for *T. cruzi* without detectable anatomic-physiological changes, being classified in the indeterminate form (Coura, 2007). The other amount may develop the cardiac, digestive or cardiodigestive forms (Rassi et al., 2012) along the years (determinate form). Chronic cardiac form is the most expressive manifestation of Chagas disease because of its frequency and severity (Rassi et al., 2009).

Chagas' disease chemotherapy is performed with benznidazole or nifurtimox, drugs that not only are genotoxic but also present severe side effects that might lead to treatment interruption (Rassi et al., 2012). Successful chemotherapy depends on several factors: the stage of disease, patient's age, and biochemical characteristics of the *T. cruzi* strain. A critical feature related to failure in chemotherapy relies on resistance mechanisms, observed in naïve or in selected strains during the treatment. Resistance to benznidazole and nifurtimox have been reported in a study evaluating 26 strains (Filardi and Brener, 1987). Additionally, the treatment with both drugs can induce resistance in distinct *T. cruzi* strains *in vitro* and *in vivo*, configuring another obstacle to the chemotherapy (Nirde et al., 1995; Dos Santos et al., 2008). Broad-spectrum resistance, named Multidrug Resistance (MDR) phenotype, is determined by cross-resistance to structurally and functionally unrelated drugs, and could be a limiting factor to the treatment of parasitosis. Among the factors related to the onset of the MDR phenotype, the best characterized is the overexpression of membrane transporters, mostly from the ATP-Binding Cassette (ABC) protein superfamily (Gottesman, 2002).

ABC proteins share the well-conserved ABC module and are divided into 8 subfamilies in eukaryotes (ABCA to ABCI) (Dassa, 2011). The “full size” transporter consists of two transmembrane domains (TMD) involved with the attachment and transport of the substrate, and two ABC modules, also known as nucleotide binding domains (NBDs), which bind and hydrolyzes ATP. The most widely studied ABC superfamily protein is ABCB1 (subfamily B, member 1), also known as P-glycoprotein (Pgp), and ABCC1 (subfamily C, member 1), also known as Multidrug Resistance Protein-1 (MRP1). Those proteins were the first cloned human ABC transporters and characterized by their ability to confer MDR phenotype to tumor cells (Juliano and Ling, 1976; Cole et al., 1992). Despite being described in tumor cells, ABC transporters are expressed in prokaryotes as well as eukaryotes, being associated to detoxification (Descoteaux et al., 1995; Raj et al., 2009; Boncoeur et al., 2012), efflux of inflammatory molecules (Leslie et al., 2005), and lipid translocation (Araujo-Santos et al., 2005; Aye et al., 2009).

ABCB1 transports molecules with no chemical, structural nor pharmacological relationship out the cell. In the general, substrates are hydrophobic and present aromatic rings and a positively charged nitrogen (Silva et al., 2015). ABCC1 mediates the efflux of a variety of physiological organic anions alone, in conjugation to glutathione (GSH), glucuronide or sulfate or in cotransport with GSH (Cole, 2014). Multidrug resistant cells present reduced accumulation of fluorescent dyes compared to sensitive cells and the use of MDR inhibitors reverts this phenotype. Rhodamine 123 (Rho 123) (Neyfakh, 1988) and carboxyfluorescein (CFDA) (van der Kolk et al., 1998) were introduced as good fluorescent substrates for ABCB1 and ABCCs respectively due to their chemical natures. Moreover, Verapamil (VP) (Tsuruo et al., 1981), Trifluoperazine (TFP) (Tsuruo et al., 1982), and Cyclosporin A (CsA) (Slater et al., 1986) were described as ABCB1 modulators, although CsA was able to modulate ABC proteins from different subfamilies. Several inhibitors of non-specific organic anion transporters such as probenecid and indomethacin are capable of inhibiting ABCCs, ABCB1 and solute carriers. The quinoline derivative MK-571 is a potent inhibitor of ABCCs proteins, having no effect on ABCB1 (Cole, 2014).

ABC transporters have been described in parasites from the Trypanosomatidae family as well. In the *Leishmania* spp. genome, four ABCB subfamily genes were identified. Two genes are ABCB1 homologs, encoding typical ABCB2 and ABCB4 proteins (Sauvage et al., 2009). Parasites transfected with the ABCB2 gene showed a reduction in the accumulation of the anticancer drug 5-fluorouracil, suggesting a role in the efflux of xenobiotics (Katakura et al., 2004). ABCB4 transporter contributed in cross-resistance to the leishmanicidal drugs miltefosine and edelfosine (Perez-Victoria et al., 2001). The two other genes from *Leishmania* show atypical structures and unknown functions (Sauvage et al., 2009). From ABCC subfamily, ABCC3 (MRPA or PGPA) protein was the first identified in *Leishmania* spp. and participates in the transport of metal-thiol conjugates to vesicles (Legare et al., 2001). Other ABCC subfamily genes described so far are ABCC2 (also known as PGPB), ABCC1 (PGPC), ABCC5 (PGPD), and ABCC4 (PGPE), neither related to the MDR phenotype (Sauvage et al., 2009). The last described member of subfamily ABCC is the ABCC7 transporter, also known as PRP1 (pentamidine resistance protein-1), because of its ability to confer resistance to pentamidine (Coelho et al., 2003). In *T. brucei*, ABCB genes have not been identified whereas two ABCC genes were functionally described, ABCC2 and ABCC6. The overexpression of these genes suggested that they are involved in resistance to melarsoprol and suramin (Shahi et al., 2002), drugs used in the chemotherapy of sleep disease.

In the *T. cruzi* genome, 27 ABC genes were identified (Leprohon et al., 2006), including tcpgp1 (Dallagiovanna et al., 1994) and tcpgp2 (Dallagiovanna et al., 1996) the first to be described. Although they belong to the ABC family, their roles in drug resistance are still controversial (Dallagiovanna et al., 1996; Murta et al., 2001; Campos et al., 2013). In 2003, Peelman et al. (2003) identified an ABCA-like transporter (named ABCA3). ABCA3 is a single copy gene expressed along the parasite life cycle, except in infective trypomastigote forms. This protein was located in the plasma membrane, flagellar pocket, and intracellular vesicles, possibly involved in vesicular trafficking (Torres et al., 2004). Recently, an ABCG-like transporter, named ABCG1, was found to be overexpressed in *T. cruzi* strains naturally resistant to benznidazole. In addition, the transfection of this transporter in CL Brener strain increased drug resistance (Zingales et al., 2015).

Considering that, drug resistance is an obstacle to the treatment of Chagas disease, ABC proteins could be considered as new molecular targets to improve therapy efficacy. Although ABC transporters have been identified in *T. cruzi* since the 90s, no functional evaluation was performed under physiologic conditions. Therefore, in the present work we evaluated the activities of ABCB1-like and ABCC-like transporters in *T. cruzi* Y strain, since we confirmed that P-glycoprotein genes belong to the ABCC subfamily in the parasite. Results in this report provide the first description of ABCC-like activity in invertebrate and vertebrate stages of the parasite life cycle and, similar to mammalians, the *T. cruzi* ABCC-like protein is a thiol transporter.

## Materials and methods

### Database searches and sequence analyses

Sequences of *T. cruzi* ABC genes were retrieved from Leprohon et al. (2006). In TriTryDB (tritrypdb.org) and GeneDB (http://www.genedb.org/Homepage) databases, gene accession numbers were updated and information concerning NCBI reference sequences, location, protein length, and gene topology in *T. cruzi* were obtained. All ABC proteins were verified for the presence of the ABC domain (accession number: PF00005). The amino acid sequence from all ABC proteins (Table 1) were collected from databases and a series of BLASTP alignments against these proteins was performed at the NCBI web site using as query sequences the proteins codified by tcpgp1 (AAC37205.1 from https://www.ncbi.nlm.nih.gov/protein/AAC09044.1) and tcpgp2 (CAA89197.1 from https://www.ncbi.nlm.nih.gov/protein/CAA89197.1).

**Table 1:** *Trypanosoma cruzi* ABC proteins.

**Nomenclature**	***T. cruzi* gene ID**	**NCBI Reference Sequences**	**Location**	**Putative protein length**	**Protein topology in *T. cruzi***	***T. cruzi* strain**	***L. major* gene ID**	***T. brucei* gene ID**
ABCA1	TcCLB.504881.50^*^	XP_809857	TcChr27	1750	[TM - NBD]_2_	CL Brener Esmeraldo-like	LmjF2.0300	
	TcCLB.510045.20^*^	XP_806887	TcChr27	967	TM - NBD	CL Brener Non-Esmeraldo-like		
ABCA2	TcCLB.507099.80 (a)	XP_817325	TcChr14	1836	[TM - NBD]_2_	CL Brener Esmeraldo-like	LmjF11.1220	-
ABCA3	TcCLB.504149.20^*^ (b)	XP_818098	TcChr27	1750	[TM - NBD]_2_	CL Brener Esmeraldo-like	LmjF11.1240	Tb927.11.6120
	TcCLB.503573.9 ^*^ (c)	XP_803907	TcChr27	967	TM - NBD	CL Brener Non-Esmeraldo-like		
ABCA4	TcCLB.507099.80 (a)	XP_817325	TcChr14	1836	[TM - NBD]_2_	CL Brener Esmeraldo-like	LmjF11.1250	-
ABCA5	TcCLB.504149.20^*^ (b)	XP_818098	TcChr27	1750	[TM - NBD]_2_	CL Brener Esmeraldo-like	LmjF11.1270	-
	TcCLB.503573.9^*^ (c)	XP_803907	TcChr27	967	TM - NBD	CL Brener Non-Esmeraldo-like		
ABCA10	TcCLB.510149.80^*^	XP_813909	TcChr36	1865	[TM - NBD]_2_	CL Brener Esmeraldo-like	LmjF29.0620	Tb927.3.3730
	TcCLB.506989.30^*^	XP_818638	TcChr36	1866	[TM - NBD]_2_	CL Brener Non-Esmeraldo-like		
ABCA11	TcCLB.511725.80	XP_818719	TcChr35	2260	[TM - NBD]2	CL Brener Non-Esmeraldo-like	-	-
ABCB1	TcCLB.507093.260	XP_820554	TcChr39	661	TM - NBD	CL Brener Esmeraldo-like	LmjF25.0530	Tb927.11.540.2
ABCB3	TcCLB.511537.8^*^	XP_806158/XP_809384	TcChr35	537	TM - NBD	CL Brener Esmeraldo-like	LmjF32.3080	Tb927.11.16930
	TcCLB.511021.70^*^	XP_811319	TcChr35	735	TM - NBD	CL Brener Non-Esmeraldo-like		
ABCC1	TcCLB.506417.10 (d) pseudogene	–	?	1577	[TM - NBD]_2_	CL Brener	LmjF23.0210	Tb927.8.2160
ABCC2	TcCLB.506417.10 (d) pseudogene	–	?	1577	[TM - NBD]_2_	CL Brener	LmjF23.0220	Tb927.8.2160
ABCC6	TcCLB.509007.99 pseudogene	–	TcChr31	1425	?	CL Brener Esmeraldo-like	LmjF31.1290	Tb927.4.4490
	TcCLB.507079.30^*^	XP_805658	TcChr31	388	NBD	CL Brener Esmeraldo-like		
	TcCLB.457101.30^*^	XP_805394	?	253	NBD	CL Brener		
	TcCLB.508965.14^*^	XP_815145	TcChr31	765	TM - NBD	CL Brener Non-Esmeraldo-like		
ABCC9	TcCLB.510231.29^*^	XP_805357	TcChr34	854	TM - NBD	CL Brener Esmeraldo-like	-	Tb927.4.2510
	TcCLB.447255.29^*^	XP_803480	TcChr34	220	NBD	CL Brener Esmeraldo-like		
	TcCLB.506559.100^*^	XP_821792	TcChr34	1472	[TM - NBD]_2_	CL Brener Non-Esmeraldo-like		
ABCD1	TcCLB.506925.530	XP_821597	TcChr39	664	TM - NBD	CL Brener Esmeraldo-like	LmjF27.0470	Tb927.11.1070
ABCD2	TcCLB.508927.20^*^	XP_804559	TcChr31	674	TM - NBD	CL Brener Esmeraldo-like	LmjF31.0540	Tb927.4.4050
	TcCLB.509237.30^*^	XP_814630	TcChr31	674	TM - NBD	CL Brener Non-Esmeraldo-like		
ABCD3	TcCLB.510431.150	XP_819234	TcChr39	635	TM - NBD	CL Brener Esmeraldo-like	LmjF33.1860	Tb927.11.3130
ABCE1	TcCLB.508637.150^*^	XP_815243	TcChr10	647	[NBD]_2_	CL Brener Non-Esmeraldo-like	LmjF21.0710	Tb927.10.1630
	TcCLB.511913.9 pseudogene	–	TcChr10	418	[NBD]_2_	CL Brener Non-Esmeraldo-like		
	TcCLB.464879.9^*^	XP_802148	TcChr10	339	[NBD]_2_	CL Brener Non-Esmeraldo-like		
ABCF1	TcCLB.504867.20^*^	XP_812776	TcChr36	723	[NBD]_2_	CL Brener Esmeraldo-like	LmjF3.0160	Tb927.10.3170
	TcCLB.510943.80^*^	XP_817081	TcChr36	723	[NBD]_2_	CL Brener Non-Esmeraldo-like		
ABCF2	TcCLB.508897.30	XP_810886	TcChr40	594	[NBD]_2_	CL Brener Non-Esmeraldo-like	LmjF19.0800	Tb927.10.15530
ABCF3	TcCLB.509105.130	XP_814891	TcChr37	673	[NBD]_2_	CL Brener Non-Esmeraldo-like	LmjF33.0310	Tb927.10.10880
ABCG1	TcCLB.506249.70^*^ (e)	XP_806666	TcChr37	665	NBD - TM	CL Brener Esmeraldo-like	LmjF06.0080	Tb927.10.7700
	TcCLB.508231.190^*^ (f)	XP_818614	TcChr37	665	NBD - TM	CL Brener Non-Esmeraldo-like		
ABCG2	TcCLB.506249.70^*^ (e)	XP_806666	TcChr37	665	NBD - TM	CL Brener Esmeraldo-like	LmjF06.0090	Tb927.10.7700
	TcCLB.508231.190^*^ (f)	XP_818614	TcChr37	665	NBD - TM	CL Brener Non-Esmeraldo-like		
ABCG3	TcCLB.506249.70^*^ (e)	XP_806666	TcChr37	665	NBD - TM	CL Brener Esmeraldo-like	LmjF06.0100	Tb927.10.7700
	TcCLB.508231.190^*^ (f)	XP_818614	TcChr37	665	NBD - TM	CL Brener Non-Esmeraldo-like		
ABCG4	TcCLB.506579.10^*^	XP_811527	TcChr7	700	NBD - TM	CL Brener Esmeraldo-like	LmjF15.0890	Tb927.9.6310
	TcCLB.507241.39^*^	XP_806410	TcChr7	290	NDB	CL Brener Non-Esmeraldo-like		
ABCG5	TcCLB.504425.70^*^	XP_813191	TcChr22	1171	NBD - TM	CL Brener Esmeraldo-like	LmjF23.0380	Tb927.8.2380
	TcCLB.509331.200^*^	XP_816786	TcChr22	1170	NBD - TM	CL Brener Non-Esmeraldo-like		
ABCG6	TcCLB.507681.100	XP_818599	TcChr4	682	NBD - TM	CL Brener Non-Esmeraldo-like	LmjF36.2890	Tb927.10.7360
ABCH1	TcCLB.510381.20^*^	XP_807302	TcChr27	303	NBD	CL Brener Esmeraldo-like	LmjF11.0040	-
	TcCLB.506905.40^*^	XP_806924	TcChr27	303	NBD	CL Brener Non-Esmeraldo-like		
ABCH2	TcCLB.509669.30^*^	XP_816112	TcChr36	318	NBD	CL Brener Esmeraldo-like	LmjF29.1640	-
	TcCLB.509617.80^*^	XP_809836	TcChr36	318	NBD	CL Brener Non-Esmeraldo-like		
ABCH3	TcCLB.511753.100^*^	XP_812902	TcChr32	496	[NBD]_2_	CL Brener Esmeraldo-like	LmjF30.1330	Tb927.6.2810
	TcCLB.511501.30^*^	XP_805965	TcChr32	502	[NBD]_2_	CL Brener Non-Esmeraldo-like		
Others	TcCLB.506529.160^*^	XP_821943	TcChr6	937	NBD	CL Brener Esmeraldo-like	LmjF12.1190	Tb927.1.4420
	TcCLB.510885.70^*^	XP_816152	TcChr6	937	NBD	CL Brener Non-Esmeraldo-like		
Others	TcCLB.508809.30^*^	XP_809200	TcChr23	1241	NBD	CL Brener Esmeraldo-like	LmjF33.3040	Tb927.2.5410
	TcCLB.506619.90^*^	XP_812627	TcChr23	1241	NBD	CL Brener Non-Esmeraldo-like		
Others	TcCLB.507105.70^*^	XP_811423	TcChr35	1027	NBD	CL Brener Esmeraldo-like	LmjF33.3260	Tb927.2.6130
	TcCLB.506817.20^*^	XP_809803	TcChr35	999	NBD	CL Brener Non-Esmeraldo-like		

*Subfamily names are following to the HUGO nomenclature, according to Leprohon et al. (2006). Gene accession numbers and other informations were retrieved from TriTrypDB (tritrypdb.org) and GeneDB database (http://www.genedb.org/Homepage). Letters after gene accession numbers indicate genes that present more than one nomenclature due to similar phylogeny to different L. major and T. brucei genes. Asterisks represent gene fragments of alleles that represent the same T. cruzi ORF. TM represents transmembrane domain and NBD, nucleotide-binding domain. Protein topology was based in the presence of TMD and NBD in putative protein sequence for each alleles*.

### Parasites

Epimastigote and trypomastigote forms of the *T. cruzi* Y strain were gently donated by Dr. Celio Freire de Lima of the Institute of Biophysics Carlos Chagas Filho from Federal University of Rio de Janeiro, Brazil.

### Culture of *T. cruzi* epimastigote forms

Y strain epimastigote forms were cultivated at 27°C in Brain and Heart Infusion medium (BHI, BD Biosciences, São Paulo, SP, Brazil) supplemented with 10% fetal bovine serum (FBS, Life Technologies of Brazil, São Paulo, SP, Brazil), 20 μg/mL folic acid (Sigma-Aldrich, São Paulo, SP, Brazil), 12 μg/mL hemin (Sigma-Aldrich) and 50 μg/mL gentamicin (Sigma-Aldrich). For subcultures, epimastigotes were collected weekly and suspended in complete BHI medium. Prior to the experiments, epimastigotes were counted with 0.08% Trypan blue solution (Sigma-Aldrich).

### Obtainment of *T. cruzi* trypomastigote forms from cell culture

Y strain metacyclic trypomastigote forms were obtained from supernatants of infected LLC-MK2 cells. These cells were kindly donated by Dr. Celio Freire de Lima. Subcultures of LLC-MK2 were performed at 37°C in a 5% CO_2_ atmosphere with RPMI 1640 medium (Sigma-Aldrich) supplemented with 2 g/L sodium bicarbonate (Sigma-Aldrich), 50 μg/mL gentamycin and 10% FBS. Cells were trypsinized by incubation at 37°C with a solution containing 1 mM EDTA (Sigma-Aldrich) and 0.25% trypsin (Life Technologies) in phosphate-buffered saline (PBS), followed by addition of PBS supplemented with 5% FBS and centrifugation at 300 × g for 5 min. The supernatant was discarded and the cells suspended in complete RPMI medium. LLC-MK2 cells were infected with trypomastigote forms. After 24 h, non-internalized parasites were removed by discarding the culture medium. Trypomastigote preparation was obtained from LLC-MK2 cells supernatant after centrifugation at 500 × g for 10 min. Next, the precipitate containing host cells was discarded and the supernatant was centrifuged at 1,000 × g for 10 min. Finally, the supernatant was discarded and the precipitate containing the trypomastigote forms was suspended in RPMI for counting.

### MTT reduction assay

The viability of the epimastigote forms was evaluated by MTT reduction assay in the presence of ABCC-like transporter modulators and in the glutathione (GSH) biosynthesis pathway inhibitor buthionine sulfoximine (BSO, Sigma-Aldrich). Briefly, 10^7^ epimastigotes/mL in complete BHI medium were distributed in 96-well culture plates at the concentrations of 2.5, 5.0, or 7.5 mM probenecid (Sigma-Aldrich); 300, 600, or 900 μM indomethacin (Sigma-Aldrich); or 100, 200, and 300 μM MK-571 (Sigma-Aldrich). After 24 h, plates were centrifuged at 1,000 × g for 10 min and the supernatant discarded. Parasites were then incubated for 4 h at 27°C with 2.5 mg/mL MTT (Sigma-Aldrich) and 0.22 mg/mL PMS (Sigma-Aldrich) in PBS supplemented with 2 g/L glucose and 10% FBS. Plates were then centrifuged and the supernatant was discarded. Formazan crystals were dissolved in DMSO (Sigma-Aldrich) and the absorbance was measured at 570 nm on a Beckman Coulter AD340 spectrophotometer (Beckman Coulter, Brea, CA, USA).

### Measurement of non-protein thiol levels in epimastigote forms

In the assay adapted from Sarkar et al. (2009), 10^6^ epimastigotes were preincubated in microtiter plates at 27°C with either 3 mM BSO in BHI medium without FBS for 48 h or 100 μM N-ethylmaleimide (NEM, Sigma-Aldrich) in PBS for 1 h. After preincubation, parasites were centrifuged at 1,000 × g for 10 min. Supernatants were discarded and parasites suspended in PBS containing 1 μM 5-chloromethylfluorescein diacetate (CMFDA, Life Technologies) for 15 min. BSO reagent was replaced during this step in order to maintain the inhibition of GSH biosynthesis, since its regeneration is rapid after withdrawal of the inhibitor (Sarkar et al., 2009). Parasites were then centrifuged, suspended in PBS and kept on ice for acquisition by flow cytometry. CMFDA dye crosses membranes through passive diffusion. It reacts with non-protein thiols inside the cell, giving rise to the thiol-conjugated methylfluorescein (TMF), which becomes fluorescent after cleavage of the acetate radical by nonspecific esterases (Barhoumi et al., 1993). The fluorescence issued by TMF was acquired by a FACSCalibur (BD Biosciences, San Jose, CA, USA) on FL1 channel. Analysis was performed using the Summit software (version 4.3, Dako Colorado, Fort Collins, CO, USA), with a gate containing at least 50,000 viable cells.

### Carboxyfluorescein efflux assay in epimastigote and trypomastigote forms

The efflux assay was adapted from Echevarria-Lima et al. (2005), in which it was divided into two 30 min steps: accumulation and efflux. Initially, 10^6^ epimastigotes were incubated in microtiter plates with 5 μM 5(6)-carboxyfluorescein diacetate (CFDA, Sigma-Aldrich), diluted in RPMI medium to allow accumulation of the dye within the cells (accumulation step). After that time, parasites were centrifuged at 1,000 × g for 10 min and then suspended in RPMI medium to allow efflux of the dye (efflux step). Next, parasites were centrifuged, suspended in PBS and kept on ice for acquisition by flow cytometry. As CMFDA, CFDA dye is derived from acetoxymethyl ester and is able to cross the cytoplasmatic membrane by passive diffusion (Breeuwer et al., 1995). After hydrolysis by nonspecific esterases in the cytosol, CFDA originates the fluorescent substrate carboxyfluorescein (CF) that is transported out of the cell by ABCC subfamily members (Rotman and Papermaster, 1966). The fluorescence from CF was acquired by a FACSCalibur on FL1 channel. Analysis was performed using the Summit software, with a gate containing at least 50,000 viable cells. The efflux assays were performed at two temperatures: 27°C (insect vector temperature) and 37°C (temperature of positive control). The index of CF efflux was calculated using the median fluorescence intensity (MFI) for CF in parasites that accumulated the dye (accumulation step) to the CF MFI in parasites that accumulated and extruded the dye (efflux step). As negative control, parasites were not exposed to the dye. As positive control, the chronic myeloid leukemia FEPS cells were used because of their ABCC1 transporter overexpression (Daflon-Yunes et al., 2013). The cells were kindly provided by Dr. Vivian Rumjanek from the Institute of Medical Biochemistry Leopoldo de Meis, Federal University of Rio de Janeiro, Brazil.

For inhibition of the ABCC-like transport, probenecid (2.5, 5.0, or 7.5 mM), indomethacin (300, 600, or 900 μM) or MK-571 (100, 200, or 300 μM) were used in the efflux assays. The inhibition index of CF efflux was calculated by the ratio between the CF MFI of the parasite in the presence of the modulator and the CF MFI in the absence of the modulator (CTL). For inhibition of ABCB1-like transport, 5 μM cyclosporin A (CsA), 10 μM verapamil (VP, Sigma-Aldrich) or 10 μM trifluoperazine (TFP, Sigma-Aldrich) were used in the CF efflux assays. CsA was kindly provided by Dr. Marcia Capella from the Institute of Biophysics of Carlos Chagas Filho, Federal University of Rio de Janeiro, Brazil. For each experiment, CF intensity histograms were divided into two areas: negative, containing 95% of control cells; positive, containing the rest of the graph. From this, the percentage of parasites in the CF+ region was shown in each treatment. The amount of inhibited parasites for each modulator was the subtraction between the percentage of CF+ parasites found in each treatment and the percentage of CF+ parasites found in the control.

For ATP depletion, epimastigotes were preincubated with 40 mM sodium azide (Sigma-Aldrich) or 1 mM iodoacetic acid (Sigma-Aldrich) for 60 min in PBS before CF efflux assays. For non-protein thiol depletion, epimastigotes were preincubated for 60 min with 100 μM NEM in PBS supplemented with 2 g/L glucose before CF efflux assays.

For trypomastigotes, 2.5 μM CFDA was employed in the efflux assay at 37°C, while the inhibitors were used at the same concentrations previously described. Fluorescence analyzes of trypomastigotes were done with at least 10,000 gated viable cells.

### Thiol-conjugated methylfluorescein efflux assay in epimastigote forms in the presence of indomethacin

10^6^ epimastigotes were incubated in microtiter plates containing 1 μM CMFDA in PBS supplemented with 2 g/mL glucose in the presence or absence of 600 μM indomethacin (accumulation step). After 30 min, the parasites were centrifuged at 1,000 × g for 10 min. The supernatant was discarded and the parasites suspended in PBS supplemented with 2 g/L glucose in the presence or absence of indomethacin (efflux step). After 30 min, parasites were centrifuged, suspended in PBS and kept on ice for acquisition by flow cytometry. The TMF efflux assay was assessed at 27 and 37°C, and acquisition was performed as previously described.

### Rhodamine 123 efflux assay in epimastigote and trypomastigote forms

Rhodamine 123 (Rho 123) is a naturally fluorescent ABCB1 substrate and able to cross biological membranes by passive diffusion (Forster et al., 2012). 10^6^ epimastigotes were incubated in 100 nM Rho 123 (Sigma-Aldrich, cat. number R8004) microtiter plates in RPMI medium in the presence or absence of CsA (2 or 50 μM), VP (10 or 50 μM) or TFP (2 or 10 μM) (accumulation step). After 30 min, the parasites were centrifuged at 1,000 × g for 10 min. The supernatant was discarded and the parasites suspended in RPMI medium in the presence or absence of the modulators (efflux step). After 30 min, parasites were then centrifuged, suspended in PBS and kept on ice for acquisition by flow cytometry. The fluorescence issued by Rho 123 was acquired by a FACSCalibur on FL1 channel. Analysis was performed using the Summit software, with a gate containing at least 50,000 viable cells. The Rho 123 efflux assay was performed at 27 and 37°C. As negative control, the parasites were not exposed to Rho 123 dye. As positive control, chronic myeloid leukemia Lucena-1 cells were used due to their overexpression of the ABCB1 transporter (Rumjanek et al., 2001). Lucena-1 cells were kindly donated by Dr. Vivian Rumjanek.

ATP depletion was performed as previously described.

For trypomastigotes, the Rho 123 efflux assay was performed at 37°C using the same substrate concentration. For ABCB1-like modulation, 50 μM CsA, or 10 μM of either VP or TFP were used. Fluorescence analyzes were performed with at least 10,000 viable cells.

### Statistical analysis

Statistical analyzes were performed using the software GraphPad Prism (GraphPad Software, San Diego, CA, USA). For two comparisons, the *t*-student or Mann–Whitey tests were used for parametric and non-parametric data, respectively. For more than two comparisons, ANOVA or Kruskal–Wallis one-way ANOVA tests were used for parametric and non-parametric data, respectively. For paired non-parametric data, the Wilcoxon or Friedman tests were performed for two and more than two comparisons respectively. The Bonferroni's post-test was used for parametric data while the Dunn's post-test was employed for non-parametric data, both for more than two comparisons. Significance values were represented by ^*^p < 0.05, ^**^p < 0.01, and ^***^p < 0.001.

## Results

### P-glycoprotein present high identity and similarity to ABCC subfamily in *T. cruzi*

Table 1 summarizes 27 genes from ABC superfamily identified in the *T. cruzi* genome. ABC proteins were named in the HUGO nomenclature according to Leprohon et al. (2006), with similar ORFs found in the genome of related parasites such as *Leishmania major* and *Trypanosoma brucei*. Dallagiovanna et al. (1994, 1996) identified the first ABC genes in *T. cruzi*. The authors showed that putative proteins from tcpgp1 and tcpgp2 genes presented high amino acid identity to the human ABCC1 and *Leishmania torentolae* MRPA rather than to human ABCB1. However, tcpgp1 and tcpgp2 were named as Pgp genes. To clarify which subfamily *T. cruzi* ABC genes belong to, a series of BLASTP alignments against all ABC proteins on Table 1 using as query sequence the protein codified by tcpgp1 (AAC09044.1) and tcpg2 (CAA89197.1) were performed (data not shown). The analysis revealed that the protein sequence from tcpgp1 and tcpgp2 present high identity and similarity to the protein sequence from TcCLB.508965.14 and TcCLB.506417.10 gene accession numbers, which correspond to the ABCC6 and ABCC1/2 in *T. cruzi*, respectively (Supplementary Table 1). For that reason, ABCC-like activity was investigated in *T. cruzi*, as well as ABCB1-like activity, which has not been described in the parasite so far.

### MK-571 inhibits the ABCC-like activity in epimastigote and trypomastigote forms

In order to evaluate ABCC-like activity, CFDA dye was used in the efflux assay in the epimastigote forms. Initially, a dot-plot of Forward Scatter × Side Scatter was performed for parasites acquired by flow cytometry (Supplementary Figure 1). A region of viable epimastigotes was delimited for the acquisition of CF fluorescence intensity histograms. The increase in both percentage of CF+ parasites and in the CF MFI after treatment with ABC pharmacological inhibitors indicated ABCC activity.

**Figure 1:**
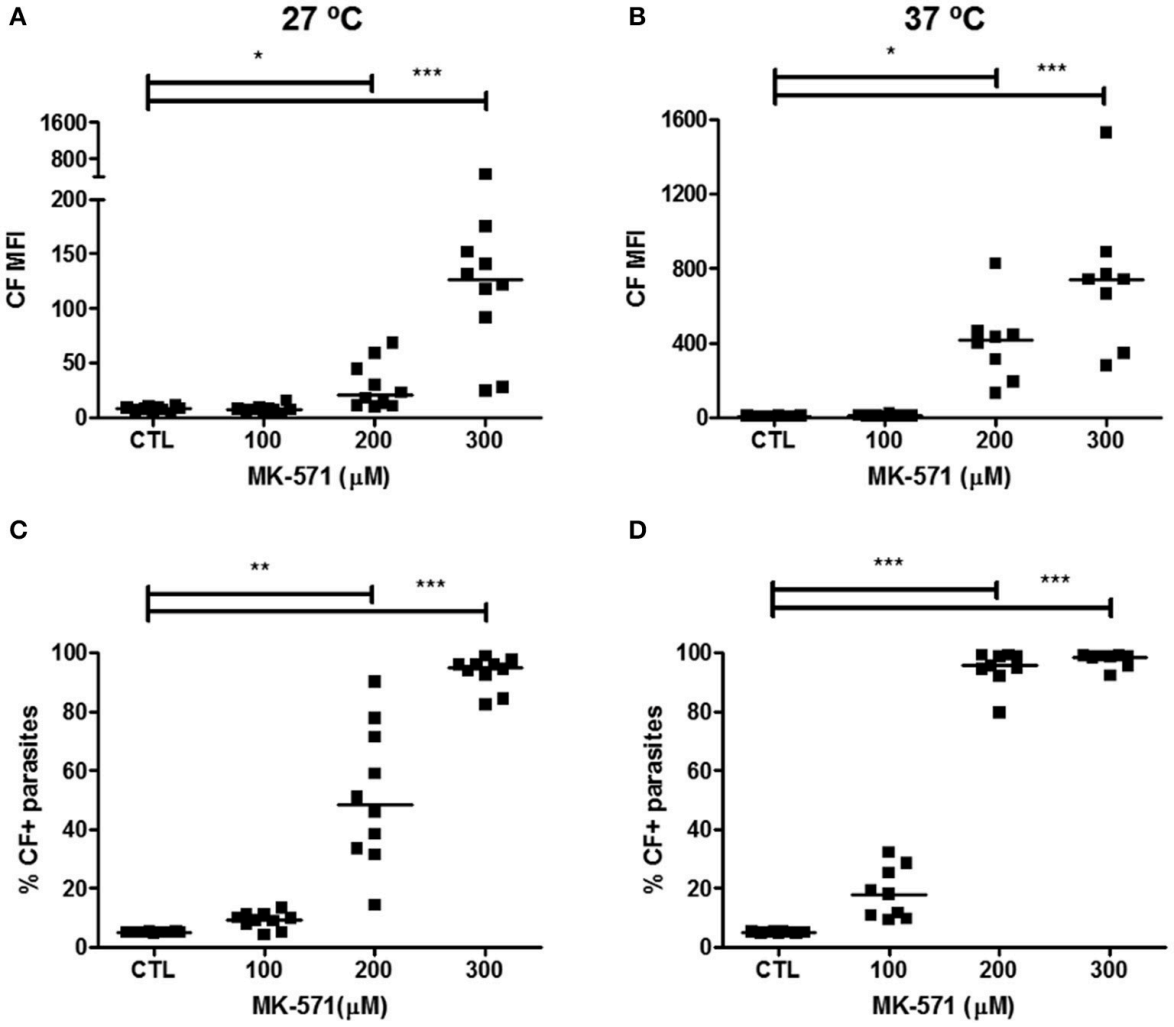
Inhibition of ABCC-like activity by MK-571 in epimastigote forms of the *T. cruzi* Y strain. ABCC-like activity was evaluated by the carboxyfluorescein (CF) efflux assay. 10^6^ epimastigotes were incubated in medium containing 5 μM CFDA in the presence or absence of 100, 200, or 300 μM MK-571 for 30 min. Parasites were then centrifuged and incubated in medium in the presence or absence of MK-571 for another 30 min. After incubation, parasites were centrifuged, suspended in PBS and kept on ice for acquisition by flow cytometry. Graphs in **(A,B)** represent the median fluorescence intensity (MFI) for CF, **(C,D)** the percentages of CF+ parasites at 27 and 37°C, respectively. The Friedman statistical test was used with *n* = 10 (27°C) and *n* = 8 (37°C) independent experiments. Lines represent the median for each group and the significance values were represented by ^*^p < 0.05; ^**^p < 0.01, and ^***^p < 0.001.

Treatment with probenecid at the concentration of 7.5 mM increased CF MFI and percentage of CF+ parasites (Supplementary Figure 2). Compared to the non-treated parasites (CTL), 5.16% of the parasites were inhibited at 27°C and 43.52% at 37°C after probenecid treatment. Indomethacin inhibited ABCC activity at the concentrations of 600 and 900 μM (Supplementary Figure 3). At 27°C, 18.14% and 37.80% of parasites were modulated with 600 and 900 μM indomethacin, respectively. At 37°C, the modulation observed was higher, of 55.34% and 85.44%, respectively. For MK-571, the specific modulator for the ABCC subfamily, 200 μM inhibited 43.23% and 90.54% of epimastigote forms at 27 and 37°C, whereas 300 μM inhibited 89.98% and 93.51% at 27 and 37°C, respectively (Figure 1). However, treatments with 900 μM indomethacin and 300 μM MK-571 induced cytotoxicity to epimastigote forms (Supplementary Figure 4). Therefore, those concentrations were excluded from the next evaluations.

**Figure 2:**
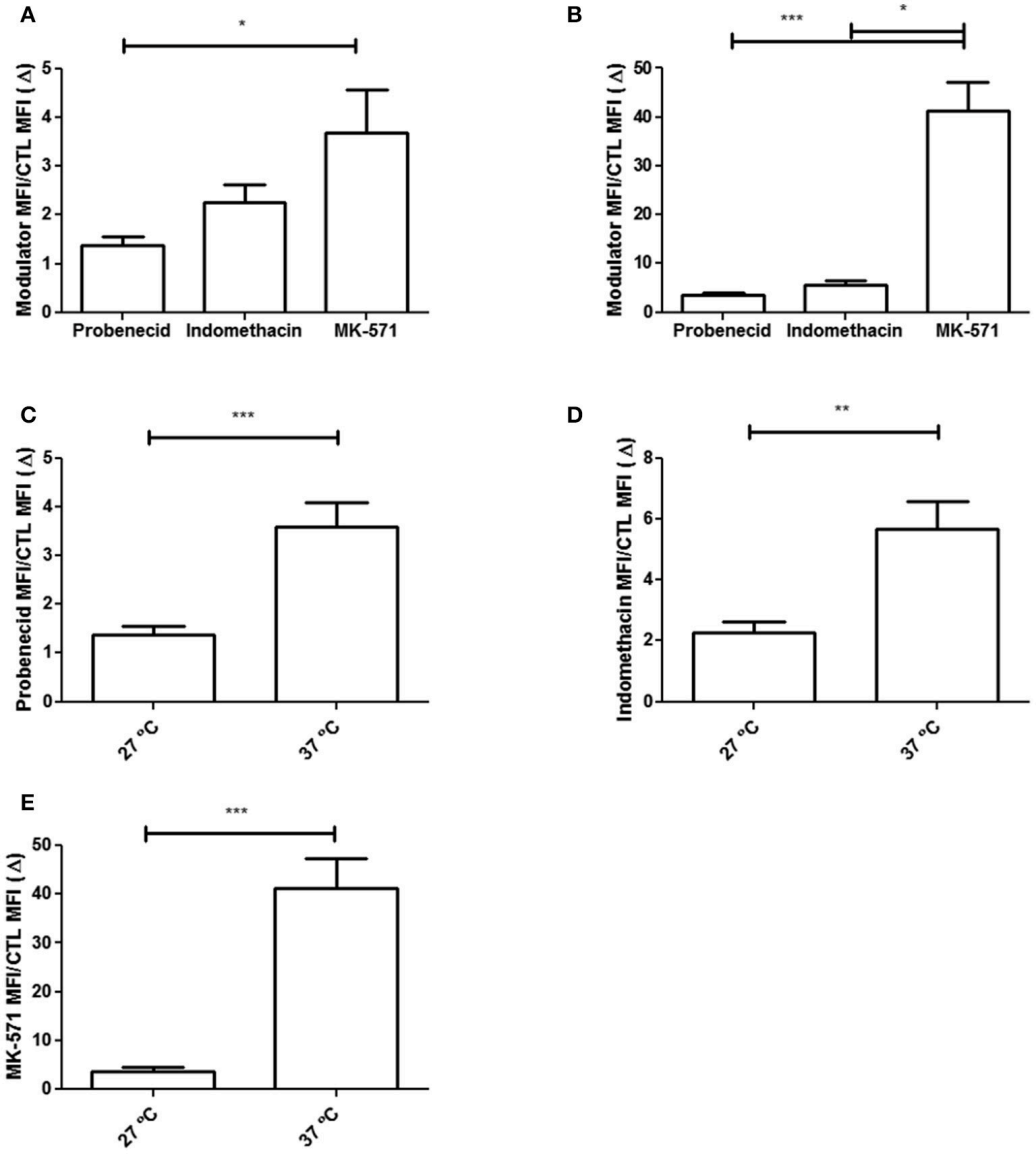
Inhibition index of CF efflux (Δ) by ABCC modulators in epimastigote forms of the *T. cruzi* Y strain. Inhibition index of CF efflux (Δ) was calculated by the ratio between the CF MFI of the parasite in the presence of the modulator and the CF MFI in the absence of the modulator (CTL). Graphs in **(A,B)** represent the Δ values in the presence of the modulators at 27 and 37°C, respectively. Comparison between Δ indexes at 27 and 37°C are shown in **(C)** for 7.5 mM probenecid, in **(D)** for 600 μM indomethacin and in **(E)** for 200 μM MK-571. The Kruskal–Wallis and **(C–E)** Mann–Whitney **(A,B)** statistical tests were performed with *n* = 10 (27°C) and *n* = 8 (37°C) independent experiments. Bars represent the mean ± standard error and the significance values were represented by ^*^p < 0.05, ^**^p < 0.01, and ^***^p < 0.001.

Comparing the subtoxic concentrations of 7.5 mM probenecid, 600 μM indomethacin and 200 μM MK-571, results showed that MK-571 was more efficient in inhibiting CF efflux in both assay temperatures. This result became evident when the CF efflux inhibition index (Δ) was evaluated (Figure 2). In addition, regardless of the modulator employed, CF efflux was higher at 37°C, indicating that temperature plays a role on the function of ABCC proteins.

**Figure 3:**
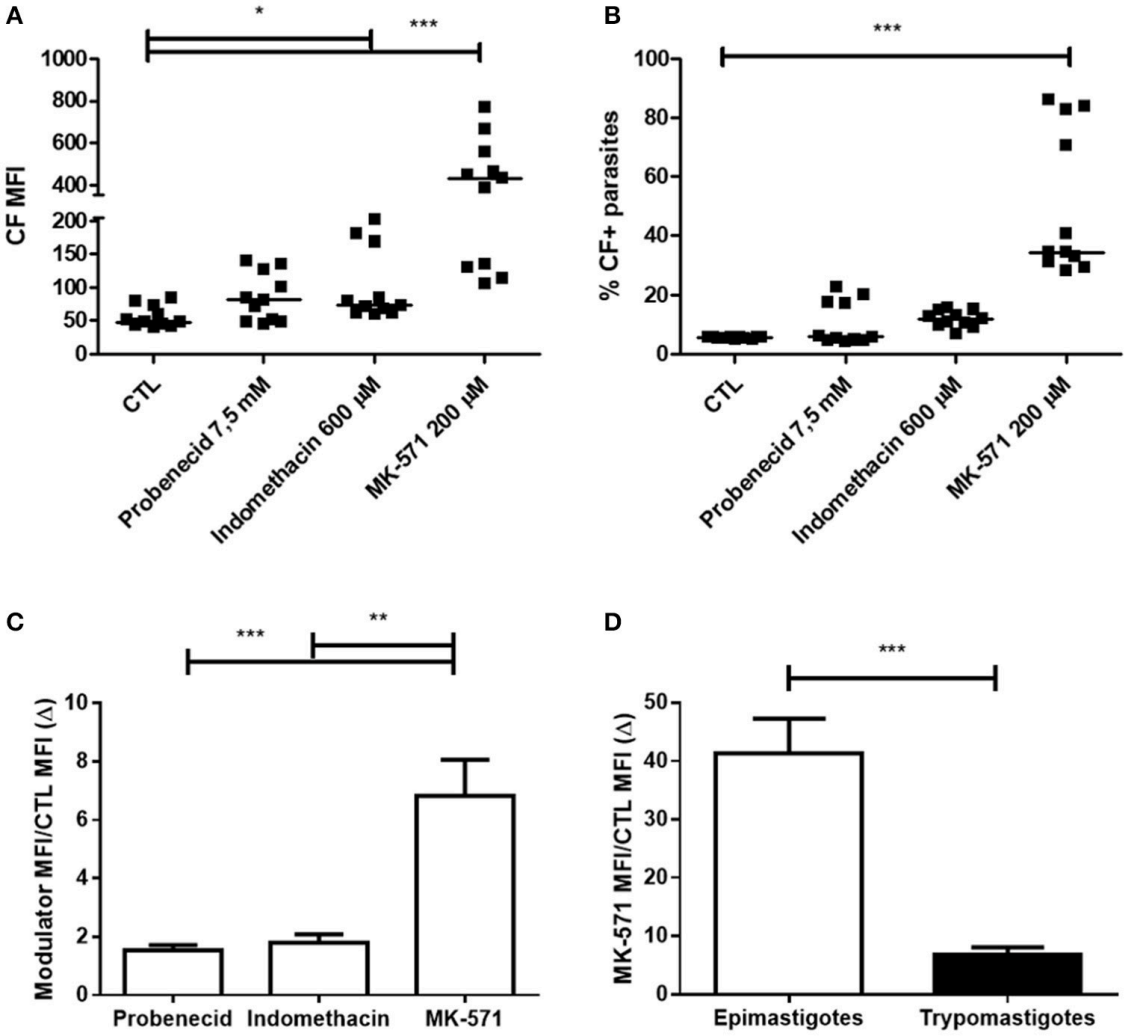
Inhibition of ABCC-like activity by MK-571 in trypomastigote forms of the *T. cruzi* Y strain. ABCC-like activity was evaluated by the carboxyfluorescein (CF) efflux assay. 10^6^ trypomastigotes were incubated in medium containing 2.5 μM CFDA in the presence or absence of 7.5 mM probenecid, 600 μM indomethacin or 200 μM MK-571 for 30 min. Parasites were then centrifuged and incubated in medium in the presence or absence of the modulators for another 30 min. After incubation, parasites were centrifuged, suspended in PBS and kept on ice for acquisition by flow cytometry. Graph in **(A)** represents the median fluorescence intensity (MFI) for CF, in **(B)** the percentages of CF+ parasites for the assay performed at 37°C. Inhibition index of CF efflux (Δ) was calculated by the ratio between the CF MFI of the parasite in the presence of the modulator and the CF MFI in the absence of the modulator (CTL). Comparison between Δ indexes are shown in the graphs in **(C)** for modulators and in **(D)** trypomastigote and epimastigote forms. The Friedman **(A–C)** and Man–Whitney **(D)** statistical test were used with *n* = 11 independent experiments. Lines represent the median for each group and the significance values were represented by ^*^p < 0.05, ^**^p < 0.01, and ^***^p < 0.001.

Trypomastigote forms also presented ABCC-like activity, which was inhibited by subtoxic concentrations of 200 μM MK-571 (Figures 3A,B). Representative dot-plots of Forward Scatter × Side Scatter and histograms for CF fluorescence in trypomastigotes are represented in Supplementary Figure 5. The percentage of inhibited trypomastigotes by MK-571 compared to control was about 29%. The inhibition index (Δ) of CF efflux for MK-571 was higher for epimastigote than trypomastigote forms, suggesting that epimastigotes present higher ABCC-like activity (Figures 3C,D).

### ATP depletion inhibits the ABCC-like activity in epimastigote forms

**Figure 4:**
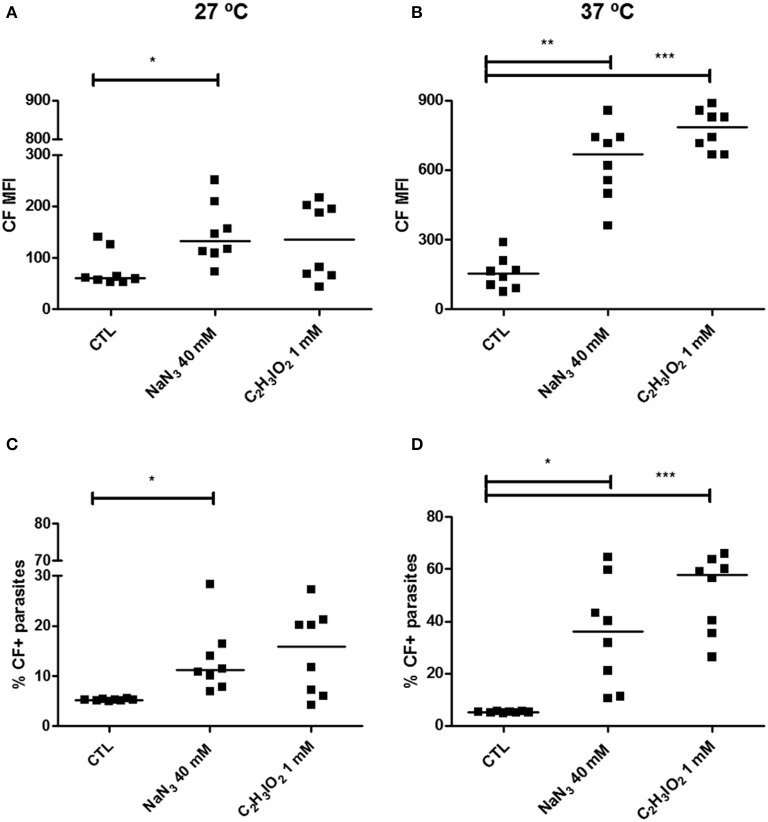
Inhibition of ABCC-like activity after ATP depletion in epimastigote forms of the *T. cruzi* Y strain. ABCC-like activity was evaluated by the carboxyfluorescein (CF) efflux assay. 10^6^ epimastigotes were preincubated for 60 min at 27 or 37°C with 40 mM sodium azide (NaN_3_) or 1 mM iodoacetic acid (C_2_H_3_IO_2_) in PBS. Parasites were then centrifuged and suspended in PBS containing 5 μM CFDA. After 30 min, parasites were centrifuged and incubated in PBS for another 30 min. After incubation, parasites were centrifuged, suspended in PBS and kept on ice for acquisition by flow cytometry. Graphs shown in **(A,B)** represent the CF MFI, **(C,D)** the percentages of CF+ parasites at 27 and 37°C, respectively. The Friedman statistical test was used with *n* = 8 independent experiments. Lines represent the median for each group and the significance values were represented by ^*^p < 0.05; ^**^p < 0.01, and ^***^p < 0.001.

To confirm whether the observed CF transport could be performed by ABCC subfamily members, CF efflux assay was evaluated in the absence of ATP (Figure 4). The percentage of inhibited parasites at 27°C was of 5.91% in the presence of sodium azide and 10.72% in iodoacetic acid. When temperature was increased to 37°C, there was a concomitant increase in the percentage of inhibited epimastigotes, which was of 30.74% after sodium azide and of 52.76% after iodoacetic acid treatments.

**Figure 5:**
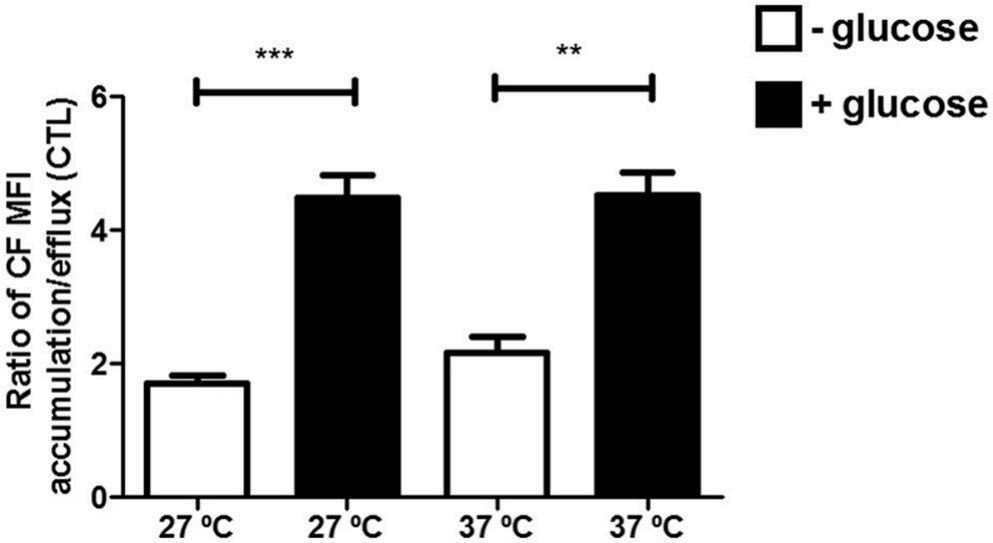
Reduced ABCC-like activity in absence of an energy source in epimastigote forms of the *T. cruzi* Y strain. ABCC-like activity was evaluated by the carboxyfluorescein (CF) efflux assay. 10^6^ epimastigotes were incubated in medium in the presence or absence of glucose containing 5 μM CFDA in the accumulation step. After 30 min, parasites were centrifuged and incubated in RPMI or PBS for another 30 min. After incubation, parasites were centrifuged, suspended in PBS and kept on ice for acquisition by flow cytometry. The Friedman statistical test was used with *n* = 8 independent experiments. Bars represent the mean ± standard error and the significance values were represented by ^**^p < 0.01 and ^***^p < 0.001.

As ABC transporters need an energy source to support the hydrolysis of ATP for their appropriate function, the CF efflux was tested in presence or absence of glucose. This dependence became clear when evaluating the efflux index for CF (Figure 5). The efflux index (MFI accumulation/efflux ratio) increased in at least two-fold when the assay was performed in presence of glucose, at both temperatures. In other words, the efflux of CF is dependent on an energy source in the assay medium and, when energy was depleted, ABCC-like activity was reduced.

### Cyclosporin A inhibits the ABCC-like activity in epimastigote forms

CsA, VP and TFP modulators are known substrates of the ABCB1 protein, and may competitively inhibit the transport of other substrates. However, some of these inhibitors may also modulate the efflux activity by ABCC members in humans. Considering this, the effect of these modulators for the CF efflux assay was evaluated in *T. cruzi* epimastigote forms. In the Supplementary Figure 6, VP and TFP did not inhibit the CF efflux at both temperatures, since less than 5% of the parasites were inhibited. The addition of CsA at 50 μM increased CF MFI and the percentage of CF+ parasites of 5% in the controls to 33.54 and 73.08% at 27 and 37°C respectively, demonstrating an inhibition of ABCC-like activity.

### Thiol depletion reduced the ABCC-like activity in epimastigote forms

**Figure 6:**
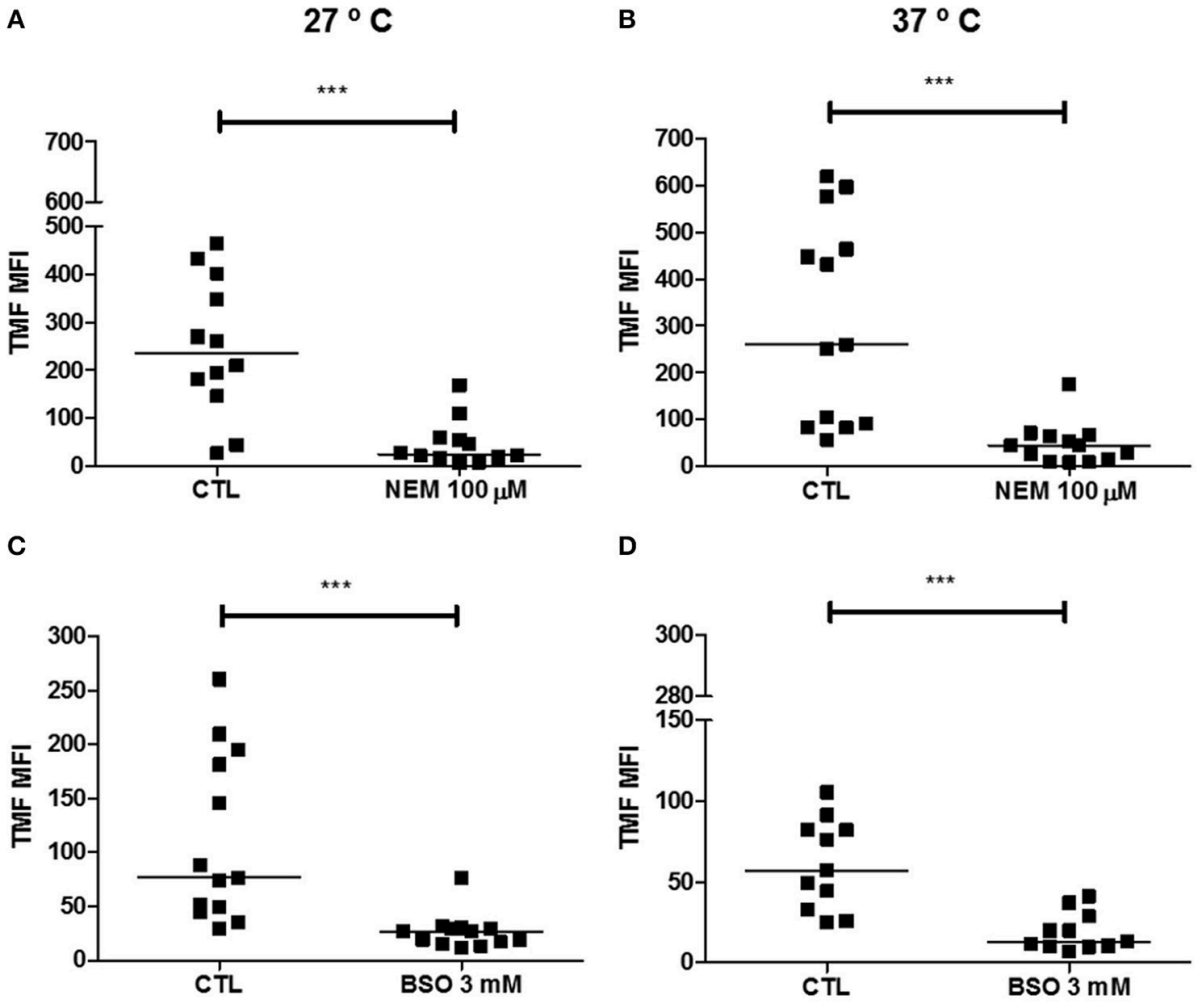
Depletion of non-protein thiols in epimastigote forms of the *T. cruzi* Y strain. 10^6^ epimastigotes were preincubated with 100 μM N-ethylmaleimide (NEM) in PBS for 60 min or with 3 mM buthionine sulfoximine (BSO) in medium for 48 h at 27°C. Then, parasites were centrifuged and suspended in PBS containing 1 μM CMFDA at 27 or 37°C. After 15 min of incubation, parasites were centrifuged, suspended in PBS and kept on ice for acquisition by flow cytometry. Graphs shown in **(A,B)** represent the median fluorescence intensity (MFI) for TMF after preincubation with NEM, **(C,D)** the TMF MFI after preincubation with BSO at 27 and 37°C, respectively. The Wilcoxon statistical test was used with 11 ≤ *n* ≤ 13 independent experiments. Lines represent the median for each group and the significance values were represented by ^***^p < 0.001.

ABCC members are able to transport organic anions and xenobiotics in cotransport or conjugated with GSH, glucuronide and sulfate. Therefore, either NEM or BSO and the CMFDA dye were used to reduce and indirectly measure the levels of GSH and trypanothione [T(SH)_2_], molecules that contain non-protein thiol radicals in *T. cruzi* (Figure 6). The alkylating agent NEM reduced thiol levels in 80-90% relative to the control at both temperatures. BSO, a specific inhibitor of GSH/T(SH)_2_ biosynthesis pathway, reduced thiol levels (TMF MFI) in 65 and 77% at 27 and 37°C, respectively, compared to control. Despite being less effective than NEM, BSO did not present cytotoxicity for *T. cruzi* epimastigotes after a 48 h-incubation (Supplementary Figure 7).

**Figure 7:**
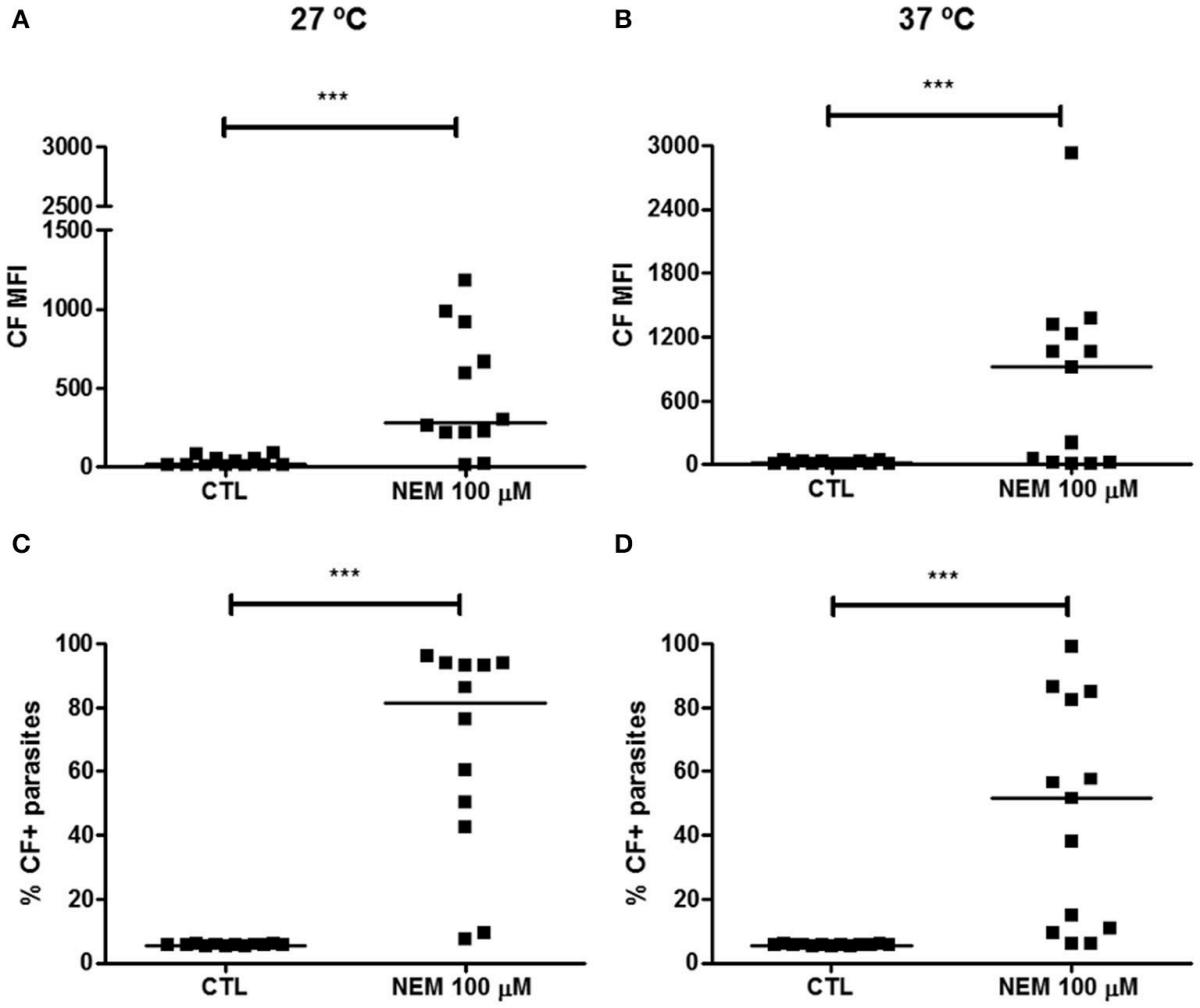
Inhibition of ABCC-like activity after depletion of non-protein thiols in epimastigote forms of the *T. cruzi* Y strain. ABCC-like activity was evaluated by the carboxyfluorescein (CF) efflux assay. 10^6^ epimastigotes preincubated in PBS for 60 min with 100 μM N-ethylmaleimide (NEM) at 27°C. Parasites were then centrifuged and suspended in PBS containing 5 μM CFDA at 27 or 37°C. After 30 min, parasites were centrifuged and incubated with PBS for another 30 min. After incubation, parasites were centrifuged, suspended in PBS and kept on ice for acquisition by flow cytometry. Graphs in **(A,B)** represent the median fluorescence intensity (MFI) for CF, **(C,D)** the percentages of CF+ parasites at 27 and 37°C, respectively. The Wilcoxon statistical test was used with *n* = 12 (27°C) and *n* = 13 (37°C) independent experiments. Lines represent the median for each group and the significance values were represented by ^***^p < 0.001.

As NEM effectively induced depletion of non-protein thiols in epimastigotes, it was used in the CF efflux assay in order to modulate the GSH/T(SH)_2_-mediated transport by ABCC members. An 1 h-preincubation with NEM increased CF MFI and percentage of CF+ parasites of 5% in the controls to 75.76% at 27°C and of 46% at 37°C (Figure 7). Results suggest that part of CF transport out of the parasite could be mediated by GSH and/or T(SH)_2_ by conjugation or cotransport, a feature of ABCC members.

### Indomethacin inhibits the ABCC-mediated efflux of thiol-conjugated compounds in epimastigote forms

**Figure 8:**
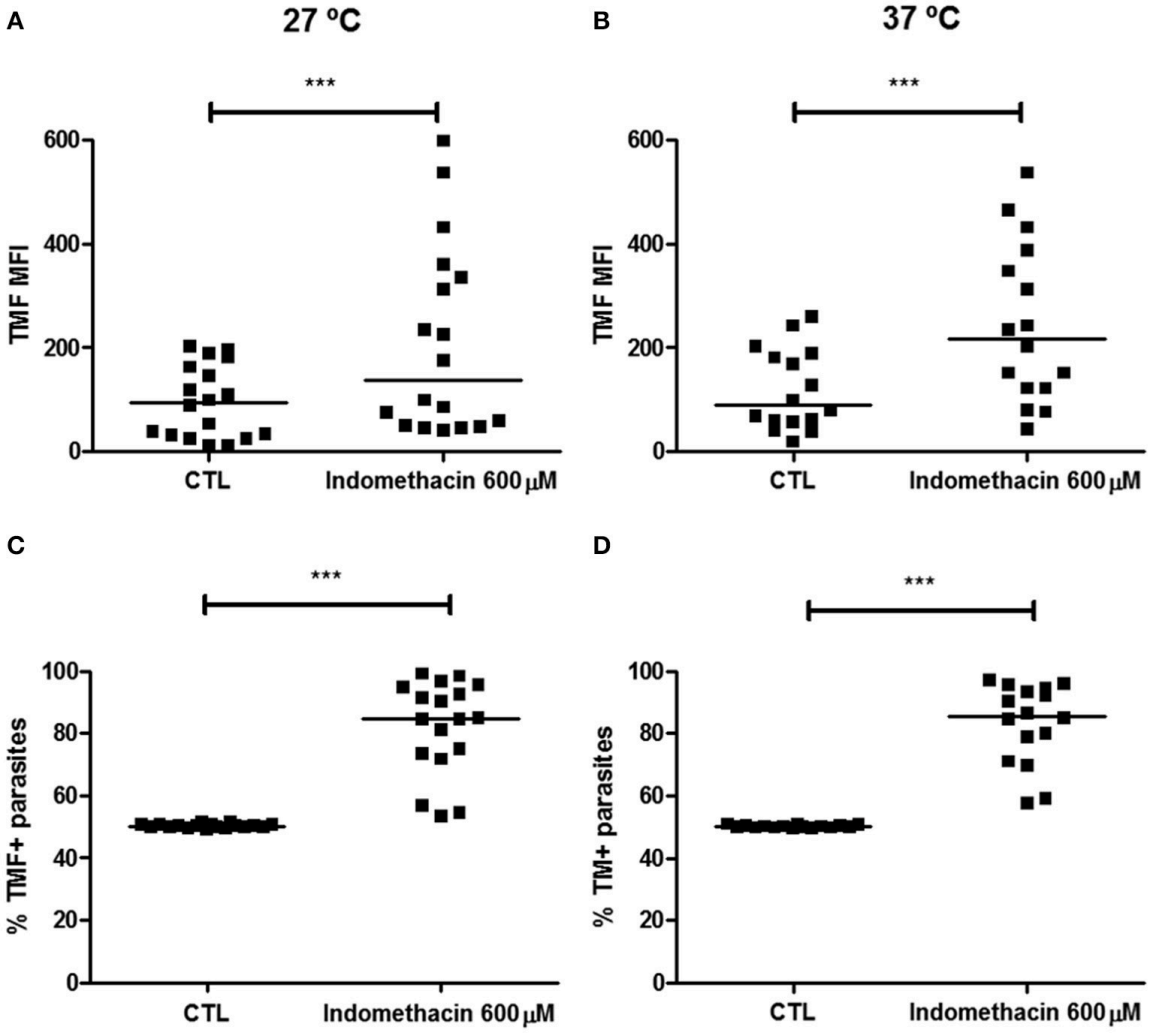
Inhibition of ABCC-mediated efflux of a thiol-conjugated compounds by indomethacin in epimastigote forms of the *T. cruzi* Y strain. ABCC-like activity was evaluated by the thiol-conjugated methylfluorescein (TMF) efflux assay. 10^6^ epimastigotes were incubated in PBS containing 1 μM CMFDA in the presence or absence of 600 μM indomethacin for 30 min. Parasites were then centrifuged and incubated in medium in the presence or absence of indomethacin for another 30 min. After incubation, parasites were centrifuged, suspended in PBS and kept on ice for acquisition by flow cytometry. Graphs in **(A,B)** represent the median fluorescence intensity (MFI) for TMF, **(C,D)** the percentages of TMF+ parasites at 27 and 37°C, respectively. The Wilcoxon statistical test was used with *n* = 18 (27°C) and 16 (37°C) independent experiments. Lines represent the median for each group and the significance values were represented by ^***^p < 0.001.

TMF is a thiol-conjugated compound since a decrease in MFI after thiol depletion was observed (Figure 6). Conversely, TMF was evaluated as a substrate for ABCC-mediated efflux in presence of indomethacin was evaluated. Representative dot-plots of Forward Scatter × Side Scatter and histograms for TMF fluorescence are found in Supplementary Figure 8. The transport of a compound conjugated to free thiol was directly inhibited by indomethacin, as observed by the increase in the TMF MFI and by inhibition of 35% of parasites compared to control at both temperatures (Figure 8).

### Epimastigote and trypomastigote forms do not present ABCB1-like activity

**Figure 9:**
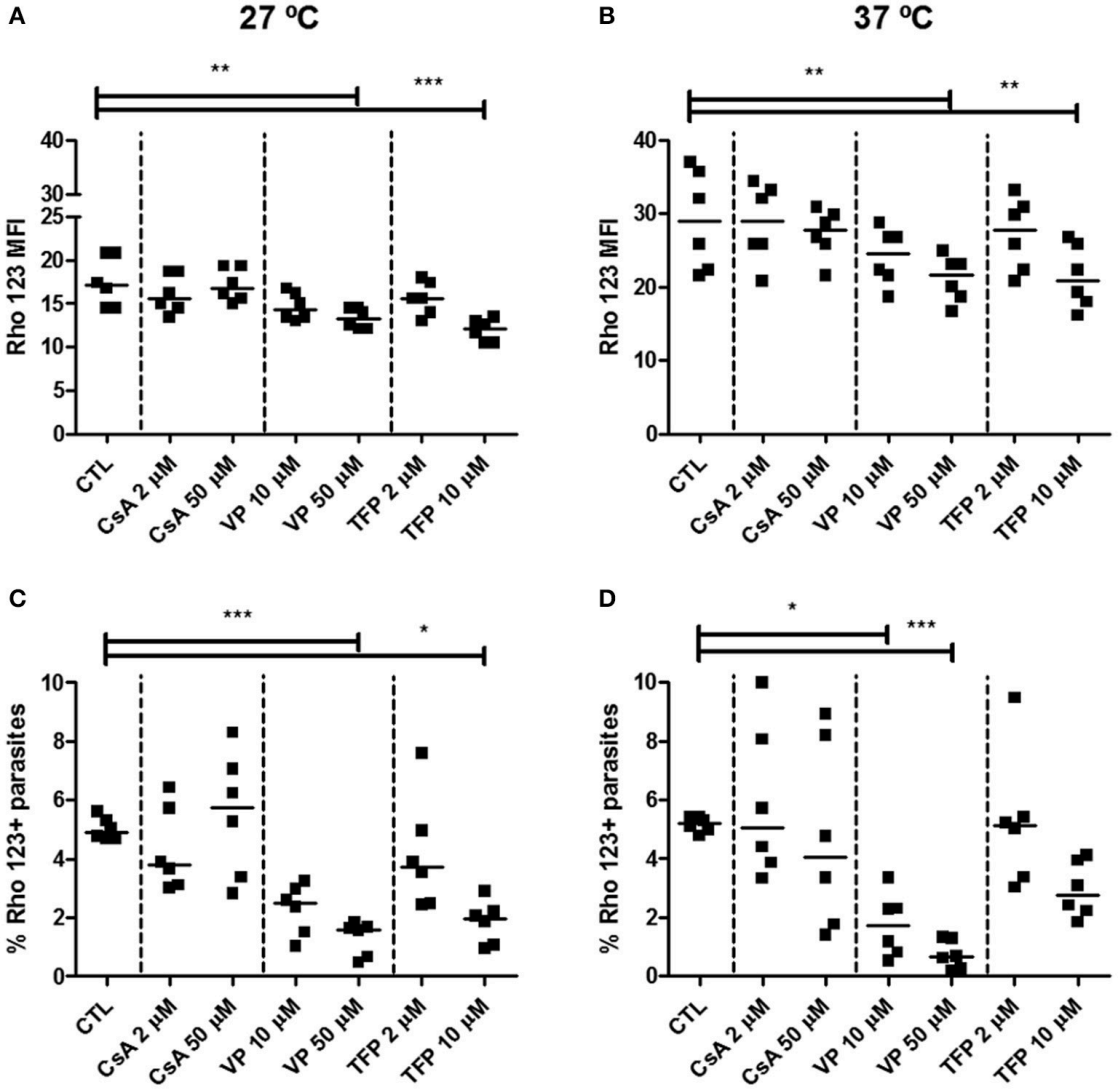
Absence of ABCB1-like activity after treatment of epimastigote forms of the *T. cruzi* Y strain with transport modulators. ABCB1-like activity was evaluated by the Rhodamine 123 (Rho 123) efflux assay. 10^6^ epimastigotes were incubated in medium containing 100 nM Rho 123 in the presence or absence of 2 or 50 μM cyclosporin A (CsA), 10 or 50 μM verapamil (VP) or 2 or 10 μM trifluoperazine (TFP) for 30 min. Parasites were then centrifuged and incubated in medium in the presence or absence of the modulators for another 30 min. After incubation, parasites were centrifuged, suspended in PBS and kept on ice for acquisition by flow cytometry. The graphs in **(A,B)** represent the median fluorescence intensity (MFI) for Rho 123, **(C,D)** the percentages of Rho 123+ parasites at 27 and 37°C, respectively. The Friedman statistical test was used with *n* = 8 independent experiments. Lines represent the median for each group and the significance values were represented by ^*^p < 0.05, ^**^p < 0.01, and ^***^p < 0.001.

**Figure 10:**
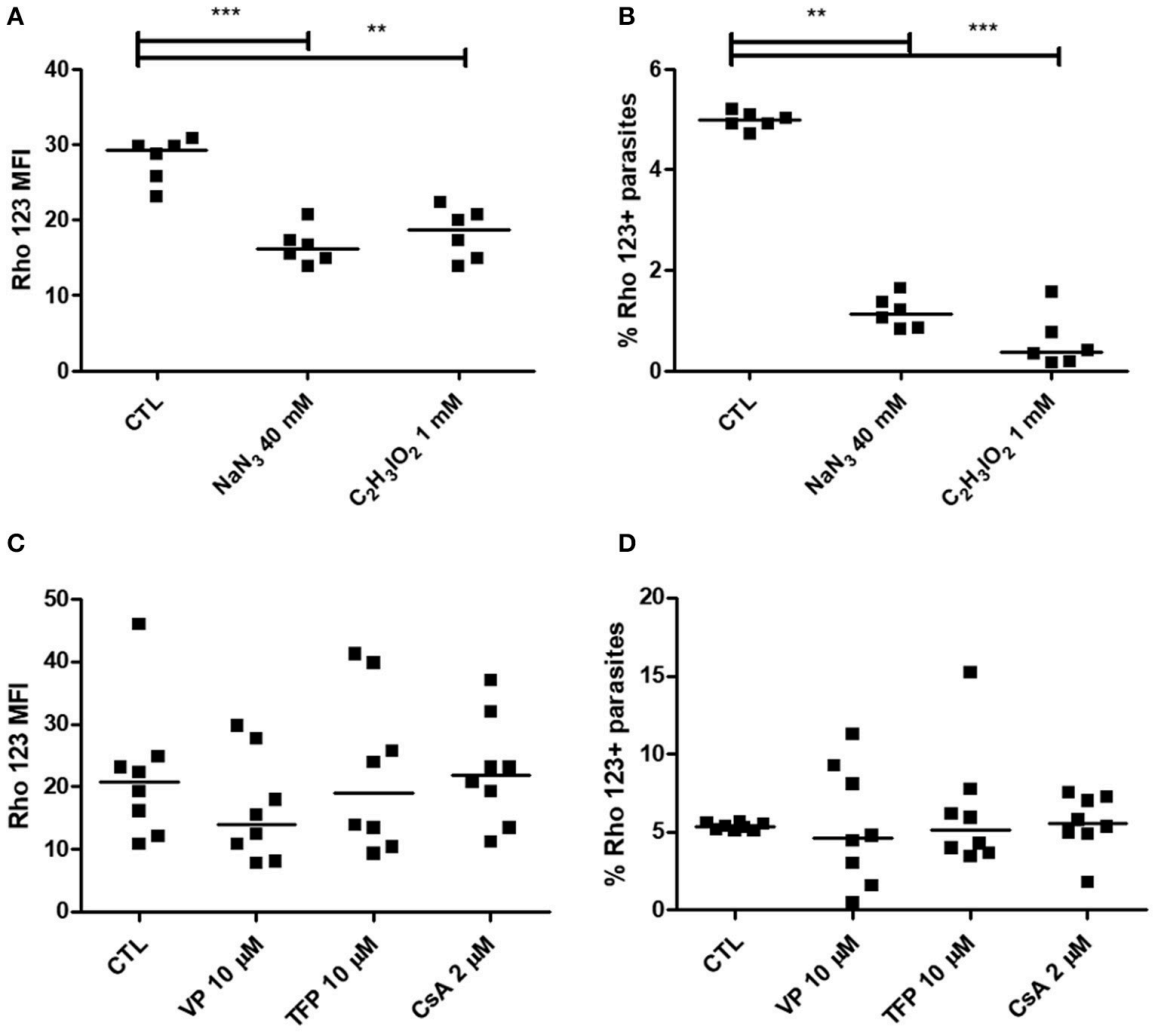
Absence of ABCB1-like activity after ATP depletion in epimastigote forms and after treatment with transport modulators in trypomastigote forms of the *T. cruzi* Y strain. ABCB1-like activity was evaluated by the Rhodamine 123 (Rho 123) efflux assay. **(A,B)** 10^6^ epimastigotes were preincubated for 60 min at 27°C with 40 mM sodium azide or 1 mM iodoacetic acid (C_2_H_3_IO_2_) in PBS. Parasites were then centrifuged and suspended in PBS containing 100 nM Rho 123. After 30 min, parasites were centrifuged and incubated in PBS for another 30 min. **(C,D)** 10^6^ trypomastigotes were incubated in medium containing 100 nM Rho 123 in the presence or absence of 2 μM cyclosporin A (CsA) or 10 μM of either verapamil (VP) or trifluoperazine (TFP) for 30 min. Parasites were then centrifuged and incubated in medium in the presence or absence of the modulators for another 30 min. After incubation, parasites were centrifuged, suspended in PBS and maintained on ice for acquisition by flow cytometry. Graphs in **(A,C)** represent the median fluorescence intensity (MFI) for Rho 123 and in **(B,D)** the percentages of Rho 123+ parasites at 37°C. The Friedman statistical test was used with *n* = 8 independent experiments. Lines represent the median for each group and the significance values were represented by ^**^p < 0.01 and ^***^p < 0.001.

In order to evaluate ABCB1-like activity, Rho 123 dye was evaluated as substrate in the efflux assay. In epimastigotes, representative dot-plots of Forward Scatter × Side Scatter and histograms for Rho 123 fluorescence can be seen in Supplementary Figure 9. Nonetheless, CsA, VP and TFP were not able to increase Rho 123 MFI nor the percentage of Rho 123+ parasites, even at 37°C (Figure 9). In some cases, Rho 123 MFI and Rho 123+ epimastigotes have decreased in the presence of modulators. Similarly, ABCB1-like activity was not detected in trypomastigote forms using the same modulators (Figures 10C,D). In trypomastigotes, representative dot-plots of Forward Scatter × Side Scatter and histograms for Rho 123 fluorescence can be seen in Supplementary Figure 10. As depicted in Figures 10A,B, after an 1 h-preincubation neither sodium azide nor iodoacetic acid were able to increase Rho 123 MFI and Rho 123+ parasites.

## Discussion

In *T. cruzi*, the first ABC genes to be described were tcpgp1 and tcpgp2, found to be similar to ABCB1 in humans and to P-glycoprotein A gene (PGPA) in *L. tarentolae*. However, the predicted protein sequences showed more identity and similarity with ABCC1, which had been discovered at that time (Dallagiovanna et al., 1994, 1996). Albeit the tcpgp1 gene had only 27.9% identity with ABCB1, it was named as Pgp. Likewise, PGPA, which was described to be similar to ABCB1, was later discovered to be closer to ABCC1. Afterwards, it was demonstrated that PGPA was able to mediate thiol efflux as well (Legare et al., 2001). Following this, it was renamed as MRPA/ABCC3, being recognized as an ABCC subfamily member (Leprohon et al., 2006). As PGPA gene in *Leishmania*, we showed that the predicted protein sequences from tcpgp1 and tcpgp2 genes correspond to ABCC6 and ABCC1/2 putative proteins described in the *T. cruzi* genome, respectively. Since ABCC-like activity has not been described in the parasite, we investigated the efflux of carboxyfluorescein (CF), a known fluorescent substrate of ABCC proteins, in live parasites.

MK-571 was the most efficient in inhibiting the CF efflux, as verified by the Δ values. It is important to note that MK-571 is the only modulator having specificity for ABCC subfamily members (Cole, 2014). Probenecid and indomethacin also inhibited the CF transport. In addition, the influence of temperature in the CF efflux was evident when the inhibition index (Δ), which was higher at 37°C regardless of the modulator used, was observed.

Trypomastigote forms of the Y strain also showed ABCC-like activity, which was inhibited by indomethacin and MK-571 in subtoxic concentrations. Although being more efficient, MK-571 inhibited about 30% of trypomastigotes, whereas in epimastigotes the inhibition was of about 90% in the same conditions. Analysis of the Δ values showed that trypomastigotes presented about five-fold less ABCC-like activity than epimastigotes, suggesting that ABCC could be regulated during the life cycle of the parasite.

ATP hydrolysis is a primordial characteristic of the ABC proteins. In the assays, CF efflux was inhibited after preincubation with ATP-depleting agents, although the modulation was lower than the one observed with MK-571. Therefore, it is possible that the concentration of sodium azide or iodoacetic acid was not sufficient to completely deplete the ATP levels, allowing the transport of CF by part of the parasite population. In epimastigotes, glucose is the preferred source for energy generation in culture; however, glucose is not abundant in the gut of the invertebrate host (Bringaud et al., 2006). Moreover, trypanosomatids do not have carbohydrate reserves, being dependent on external energy sources (Maugeri et al., 2011). Consequently, removal of glucose from the assay medium significantly reduced the CF efflux, ruling out the possibility of the transport being exclusively passive. Additionally, solute carriers like OATP1B1 are able to carry organic anions through membranes in humans, though this kind of transport is not dependent of ATP directly.

In our work, modulators employed for inhibiting ABCB1-mediated transport such as VP did not present effect on the CF efflux, while TFP was discarded as an efficient inhibitor in *T. cruzi*. On the other hand, the broad-spectrum modulator CsA was able to inhibit the ABCC-like activity in *T. cruzi* as seen in mammal cells.

An important feature of ABCC subfamily members is the ability to transport endo- and xenobiotics in cotransport or in conjugation with GSH. NEM is a sulfhydryl radical alkylating agent, being able to bind covalently to molecules containing non-protein thiols, preventing the participation of these molecules in enzymatic reactions. BSO is the specific inhibitor of γ-glutamylcysteine synthetase, an enzyme of the GSH biosynthesis pathway. In mammalians, GSH is the main non-protein thiol and plays an important role in the biotransformation of molecules and defense against electrophilic compounds (Pisoschi and Pop, 2015). In trypanosomatids, the main non-protein thiol is represented by T(SH)_2_, which is formed by two GSH molecules bound by a spermidine chain (Olin-Sandoval et al., 2010). Thus, the use of NEM masks any molecule containing an apparent thiol radical while BSO specifically reduces the synthesis of GSH and, thereafter, T(SH)_2_. Preincubation with NEM or BSO reduced non-protein thiol levels of epimastigote forms, as observed by the reduction of the TMF MFI, the thiol-conjugated product of the reaction of CMFDA with a free thiol radical. Considering this, CMFDA dye was used to indirectly measure GSH/T(SH)_2_ levels of the parasite. Results indicated that NEM was more effective in reducing non-protein thiol levels, presenting nearly 90% reduction in TMF MFI. Furthermore, the treatment with NEM inhibited the CF efflux, suggesting that part of the CF organic anion transport could be performed via cotransport with GSH or T(SH)_2_. Besides that, the TMF efflux was modulated by indomethacin, inhibiting about 35% of the parasites. The lower inhibition of TMF transport compared to CF may be due to the reduced affinity of ABCC members to thiols (Slot et al., 2011). A study carried out on vesicles enriched with different ABCC proteins demonstrated that the transport of dichlorocarboxifluorescein, analogous to CMFDA, differed between ABCC members in tumor cells (Pratt et al., 2006). Likewise, it is known that human ABCC members can transport GSH even if the GSH affinity varies among them. Regardless of the affinity, it is important to focus on that *T. cruzi* Y strain performed thiol efflux, and this kind of transport is on par with the mechanism described for ABCC members.

In both evolutionary forms neither CsA, VP, nor TFP, all used as ABCB modulators, inhibited Rho 123 efflux, since there was no increase in Rho 123 MFI nor in the percentage of Rho 123+ parasites. Results were similar when temperature was increased from 27 to 37°C or after ATP depletion. In addition, there was no difference in the Rho 123 MFI from accumulation and efflux step (data not shown). These results, together with the predicted protein sequences, suggest that the expression of an ABCB1-like transporter in the cytoplasmic membrane is very low, or even absent.

The presence of higher ABCC-like activity in epimastigote compared to trypomastigote forms could relate to the protection from microenvironment-induced oxidative stress. Hemoglobin degradation in the hematophagous insect gut releases large amounts of heme (Graca-Souza et al., 2006). Heme catalyzes many oxidation processes in biological systems, involved in the cellular respiration, metabolism, growth, and cell differentiation, which are essential processes for survival (Ciccarelli et al., 2007; Paes et al., 2011). Although it is important for *T. cruzi* growth, heme is toxic due to its ability to generate reactive oxygen species (Kumar and Bandyopadhyay, 2005), and owing to its amphipathic characteristics, is capable to associate with membrane lipids thus altering cellular permeability (Schmitt et al., 1993). Under these conditions, *T. cruzi* needs to adjust for not only changes in the potentially reducing microenvironment, but also for the increased production of reactive oxygen species that follows heme metabolism. In the most cells, antioxidative machinery relies on GSH as electron source to reduce and inactivate reactive oxygen and nitrogen species, which is regenerated by glutathione reductase (Pisoschi and Pop, 2015). *T. cruzi* produces both GSH and T(SH)_2_ (Krauth-Siegel and Comini, 2008). Notwithstanding, the parasite does not express glutathione reductase, making the antioxidant system exclusively reliant on T(SH)_2_ regeneration (Olin-Sandoval et al., 2010). In that context, the ABCC activity could transport oxidized GSH or T(SH)_2_, and also metabolites, contributing to the parasite defense system by an alternative route to T(SH)_2_ regeneration. In addition, ABCC is involved in the bioavailability of various endobiotics that could contribute for parasite survival in the host, indicating the importance of the study of its physiologic substrates.

There are three ABCC subfamily genes identified in the *T. cruzi* genome, which may be associated to ABCC-like activity observed in this study. ABCC1/2 is recognized as a pseudogene (Leprohon et al., 2006). Therefore, this transporter is not supposed to codify a protein, although it could be transcribed into mRNA. ABCC6 presents an atypical structure, lacking the sequences corresponding to the 11th and 12th transmembrane helices of TMD and the second NBD, due to the insertion of a retrotransposon (Dallagiovanna et al., 1996). In fact, functional transporters with atypical structures are described in the literature as the case of human ABCG2 (Taylor et al., 2017), and many others in parasites, as *Plasmodium falciparum* ABCB3 (Sauvage et al., 2009), which can function as an homodimer. The last ABCC subfamily gene is ABCC9, whose function is unknown in *T. cruzi*. In humans, this protein is not involved in the drug resistance and constitutes the ATP-sensitive potassium channels (Solbach et al., 2006). Conversely, the participation of ABCC9 in the transport of thiol as observed in this study cannot be ruled out, since ABCC9 could exert different functions in the parasite.

To date, there are no studies evaluating the protein expression of neither tcpgp1 nor tcpgp2. Regarding transcript levels, few papers have addressed this question. Northern blot analysis with a tcpgp1 probe revealed that a mRNA molecule that hybridizes to tcpgp1 is constitutively expressed in Y, Tulahuen and Maracay *T. cruzi* strains (Dallagiovanna et al., 1994). Subsequently, tcpgp2 was discovered in *T. cruzi* and, again by Northern Blot analysis, its mRNA was found to be expressed in amastigote and epimastigote forms. Despite not being successful in *T. cruzi*, transfection of the tcpgp2 gene in *L. tropica* resulted in mRNA expression for tcpgp2, but this was not sufficient to confer cross-resistance to different drugs. Nonetheless, resistance to benznidazole increased in transfected parasites when their EC_50_ values were compared (Dallagiovanna et al., 1996). In 1998, Murta *et al*. tested a gene probe for tcpgp in 27 *T. cruzi* strains sensitive or naturally resistant to benznidazole or nifurtimox. Authors observed the presence of polymorphism in pgp almost exclusively in the sensitive strains. Then, those 27 strains, as well as *in vitro* induced or *in vivo* selected benznidazole-resistant strains were analyzed and none presented different transcript levels for tcpgp1 and tcpgp2 (Murta et al., 2001). By differential gene expression, the same group did not observe involvement of ABC genes in the MDR phenotype of different *T. cruzi* strains (Murta et al., 2008). Campos et al. (2013) then induced resistance to benznidazole and a thiosemicarbazone derivative in the Y strain *in vitro*. The authors demonstrated rhodamine 123 efflux, which was inhibited by ABCB1 modulators, and increased ATPase activity in the resistant parasites. In addition, authors showed increased transcript levels of tcpgp1 and tcpgp2 in resistant Y parasites compared to the parental strain, although these genes are more related to subfamily C rather than B, as observed in our work. As ABC proteins are involved in drug transport in humans and other trypanosomatids, it is plausible to suggest that ABCC activity could also relate to chemotherapy resistance. Considering this, the ABC protein subfamilies rise as targets for studies aiming to better understand the mechanisms employed by *T. cruzi* to adapt to microenvironment stress. Further research is pivotal to reveal the role of ABCC transporters in the parasite physiology, during drug resistance and, ultimately, to develop new strategies for the treatment of Chagas disease.

## Author contributions

KMC: Designed research and performed the experiments, analyzed, interpreted the results, and drafted the manuscript; RCV: Designed research, contributed to the interpretation of the results, and revised the manuscript; ES: Helped in carrying out MTT reduction assay, interpreted the results, and drafted the manuscript; LBG: Helped in initial analysis of BLAST alignment and revised the manuscript; LFL, LMP, and JOP: Designed research and revised the manuscript. All authors read and approved the final version of the manuscript.

### Conflict of interest statement

The authors declare that the research was conducted in the absence of any commercial or financial relationships that could be construed as a potential conflict of interest.

## Abbreviations

Δ: Inhibition index
ABC: ATP-binding cassete
ABCB1: Subfamily B, member 1
BSO: Buthionine sulfoximine
CF: Carboxyfluorescein
CFDA: 5(6)-carboxyfluorescein diacetate
CMFDA: 5-chloromethylfluorescein
CsA: Cyclosporin A
GSH: Glutathione
MDR: Multidrug resistance
MFI: Median fluorescence intensity
MRP1: Multidrug resistance protein-1
NBD: Nucleotide binding domain
NEM: N-ethylmaleimide
Pgp: P-glycoprotein
Rho 123: Rhodamine 123
T(SH)_2_: Trypanothione
TFP: Trifluoperazine
TMD: Transmembrane domain
TMF: thiol-conjugated methylfluorescein
VP: Verapamil.
