# Supplementary material for: Functional Characterization of ABCC Proteins from Trypanosoma cruzi and Their Involvement with Thiol Transport
Source: Front Microbiol. 2018 Feb 14;9:205. doi: 10.3389/fmicb.2018.00205 (PMC5817095; doi:10.3389/fmicb.2018.00205)
Supplement: Supplementary file 1 [file Presentation1.pptx]

## Slide 1
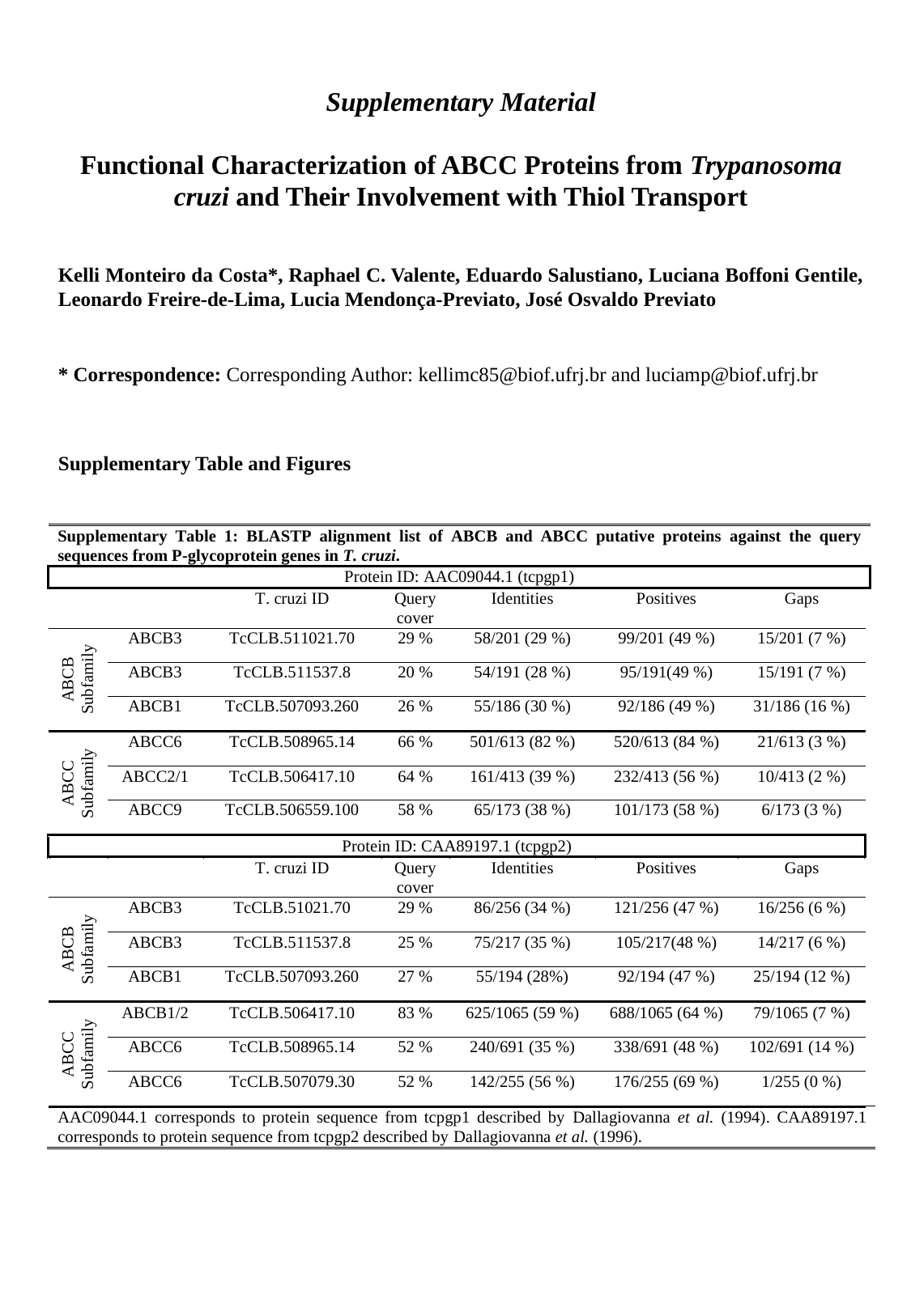

Supplementary Material
Functional Characterization of ABCC Proteins from Trypanosoma cruzi and Their Involvement with Thiol Transport
Kelli Monteiro da Costa*, Raphael C. Valente, Eduardo Salustiano, Luciana Boffoni Gentile, Leonardo Freire-de-Lima, Lucia Mendonça-Previato, José Osvaldo Previato
* Correspondence: Corresponding Author: kellimc85@biof.ufrj.br and luciamp@biof.ufrj.br
Supplementary Table and Figures

## Slide 2
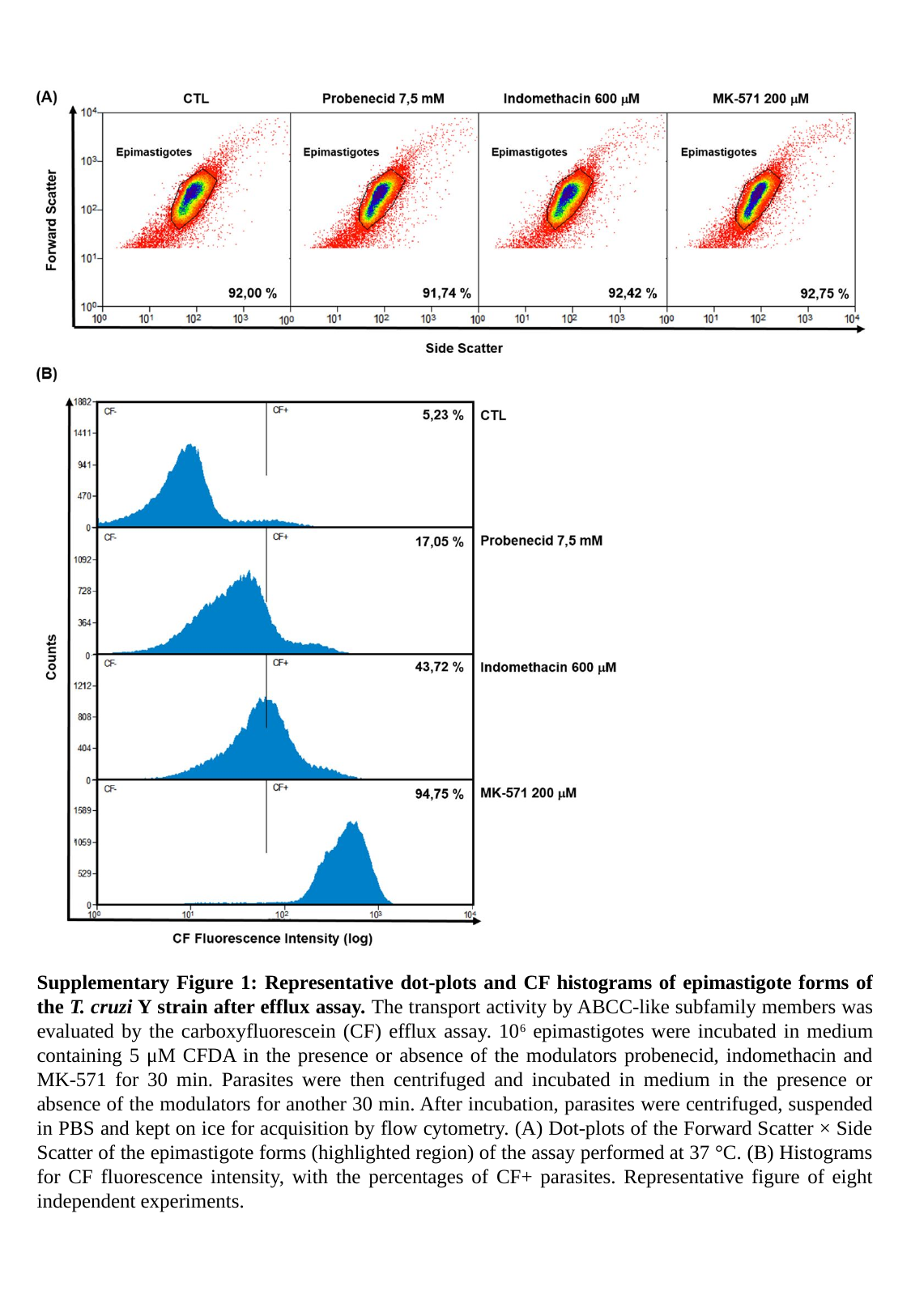

Supplementary Figure 1: Representative dot-plots and CF histograms of epimastigote forms of the T. cruzi Y strain after efflux assay. The transport activity by ABCC-like subfamily members was evaluated by the carboxyfluorescein (CF) efflux assay. 106 epimastigotes were incubated in medium containing 5 μM CFDA in the presence or absence of the modulators probenecid, indomethacin and MK-571 for 30 min. Parasites were then centrifuged and incubated in medium in the presence or absence of the modulators for another 30 min. After incubation, parasites were centrifuged, suspended in PBS and kept on ice for acquisition by flow cytometry. (A) Dot-plots of the Forward Scatter × Side Scatter of the epimastigote forms (highlighted region) of the assay performed at 37 °C. (B) Histograms for CF fluorescence intensity, with the percentages of CF+ parasites. Representative figure of eight independent experiments.

## Slide 3
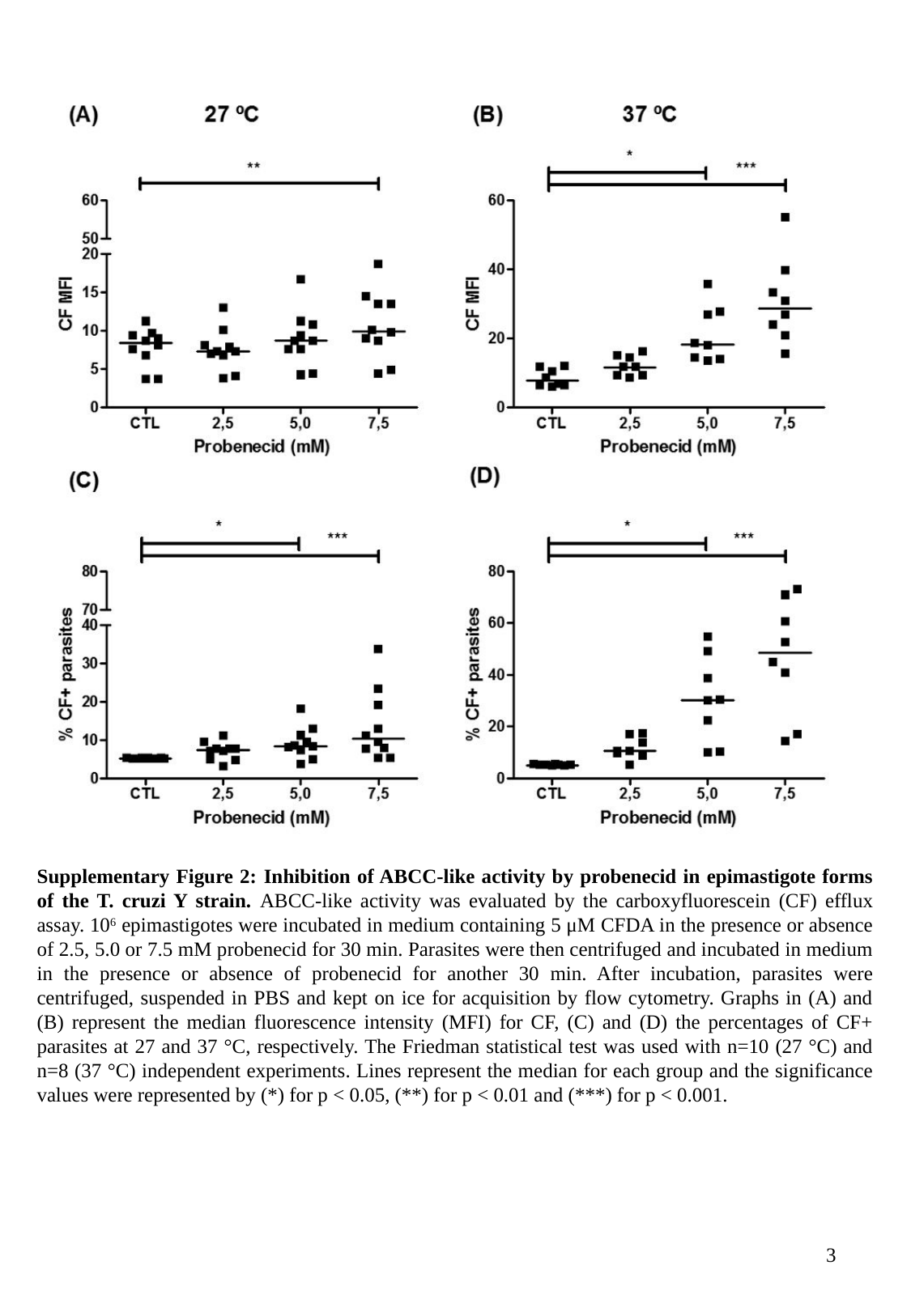

Supplementary Figure 2: Inhibition of ABCC-like activity by probenecid in epimastigote forms of the T. cruzi Y strain. ABCC-like activity was evaluated by the carboxyfluorescein (CF) efflux assay. 106 epimastigotes were incubated in medium containing 5 μM CFDA in the presence or absence of 2.5, 5.0 or 7.5 mM probenecid for 30 min. Parasites were then centrifuged and incubated in medium in the presence or absence of probenecid for another 30 min. After incubation, parasites were centrifuged, suspended in PBS and kept on ice for acquisition by flow cytometry. Graphs in (A) and (B) represent the median fluorescence intensity (MFI) for CF, (C) and (D) the percentages of CF+ parasites at 27 and 37 °C, respectively. The Friedman statistical test was used with n=10 (27 °C) and n=8 (37 °C) independent experiments. Lines represent the median for each group and the significance values were represented by (*) for p < 0.05, (**) for p < 0.01 and (***) for p < 0.001.
3

## Slide 4
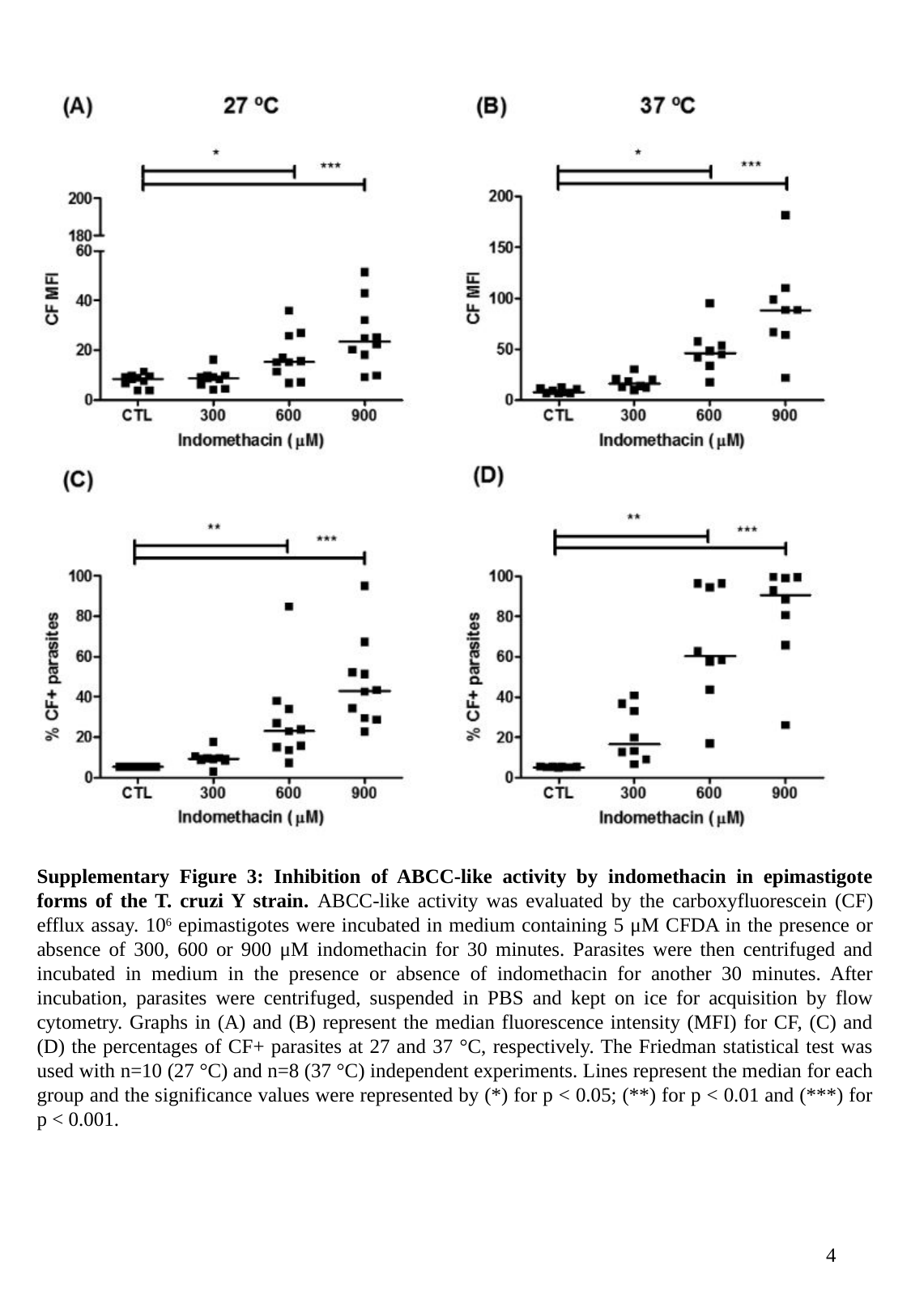

Supplementary Figure 3: Inhibition of ABCC-like activity by indomethacin in epimastigote forms of the T. cruzi Y strain. ABCC-like activity was evaluated by the carboxyfluorescein (CF) efflux assay. 106 epimastigotes were incubated in medium containing 5 μM CFDA in the presence or absence of 300, 600 or 900 μM indomethacin for 30 minutes. Parasites were then centrifuged and incubated in medium in the presence or absence of indomethacin for another 30 minutes. After incubation, parasites were centrifuged, suspended in PBS and kept on ice for acquisition by flow cytometry. Graphs in (A) and (B) represent the median fluorescence intensity (MFI) for CF, (C) and (D) the percentages of CF+ parasites at 27 and 37 °C, respectively. The Friedman statistical test was used with n=10 (27 °C) and n=8 (37 °C) independent experiments. Lines represent the median for each group and the significance values were represented by (*) for p < 0.05; (**) for p < 0.01 and (***) for p < 0.001.
4

## Slide 5
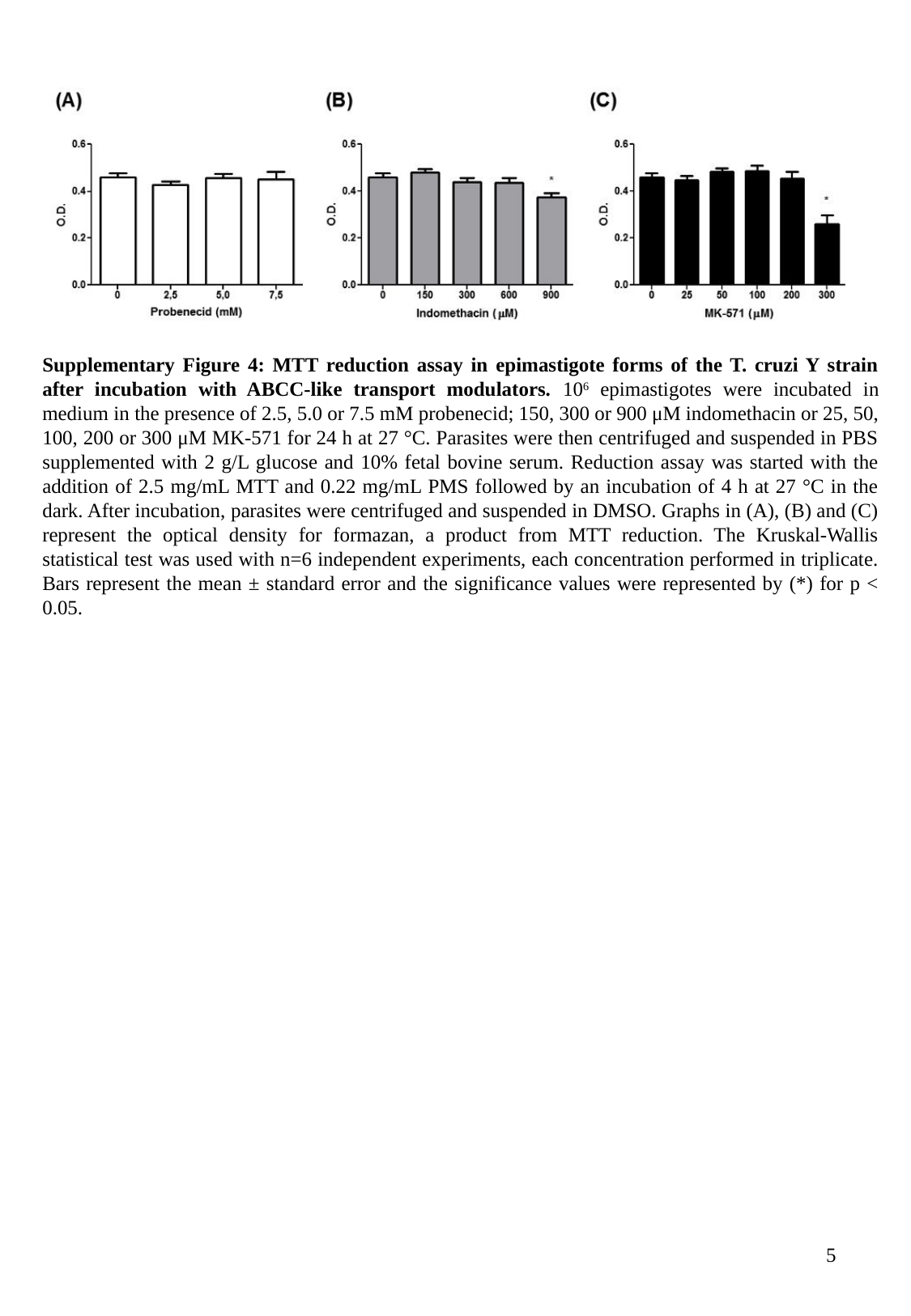

Supplementary Figure 4: MTT reduction assay in epimastigote forms of the T. cruzi Y strain after incubation with ABCC-like transport modulators. 106 epimastigotes were incubated in medium in the presence of 2.5, 5.0 or 7.5 mM probenecid; 150, 300 or 900 μM indomethacin or 25, 50, 100, 200 or 300 μM MK-571 for 24 h at 27 °C. Parasites were then centrifuged and suspended in PBS supplemented with 2 g/L glucose and 10% fetal bovine serum. Reduction assay was started with the addition of 2.5 mg/mL MTT and 0.22 mg/mL PMS followed by an incubation of 4 h at 27 °C in the dark. After incubation, parasites were centrifuged and suspended in DMSO. Graphs in (A), (B) and (C) represent the optical density for formazan, a product from MTT reduction. The Kruskal-Wallis statistical test was used with n=6 independent experiments, each concentration performed in triplicate. Bars represent the mean ± standard error and the significance values were represented by (*) for p < 0.05.
5

## Slide 6
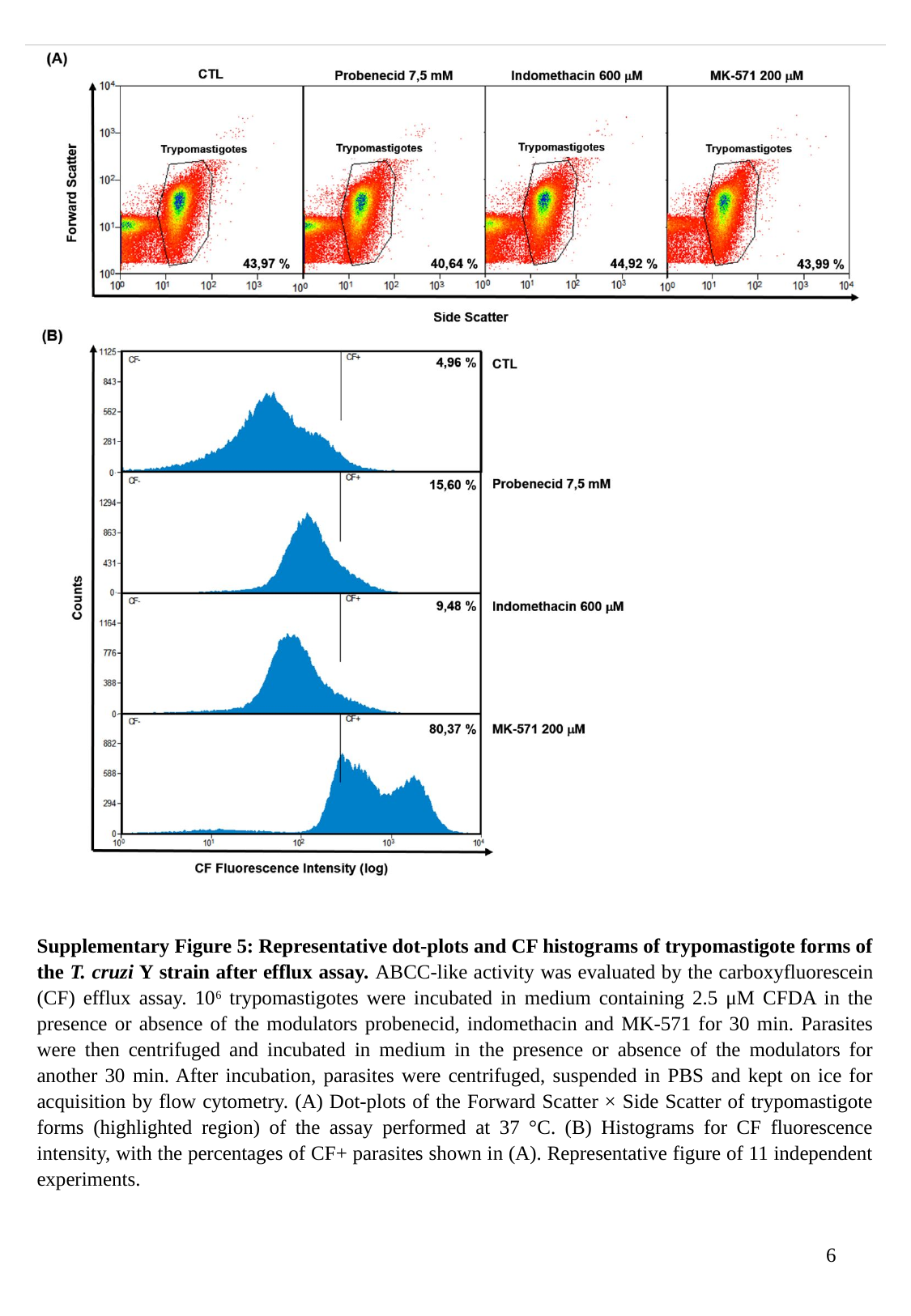

Supplementary Figure 5: Representative dot-plots and CF histograms of trypomastigote forms of the T. cruzi Y strain after efflux assay. ABCC-like activity was evaluated by the carboxyfluorescein (CF) efflux assay. 106 trypomastigotes were incubated in medium containing 2.5 μM CFDA in the presence or absence of the modulators probenecid, indomethacin and MK-571 for 30 min. Parasites were then centrifuged and incubated in medium in the presence or absence of the modulators for another 30 min. After incubation, parasites were centrifuged, suspended in PBS and kept on ice for acquisition by flow cytometry. (A) Dot-plots of the Forward Scatter × Side Scatter of trypomastigote forms (highlighted region) of the assay performed at 37 °C. (B) Histograms for CF fluorescence intensity, with the percentages of CF+ parasites shown in (A). Representative figure of 11 independent experiments.
6

## Slide 7
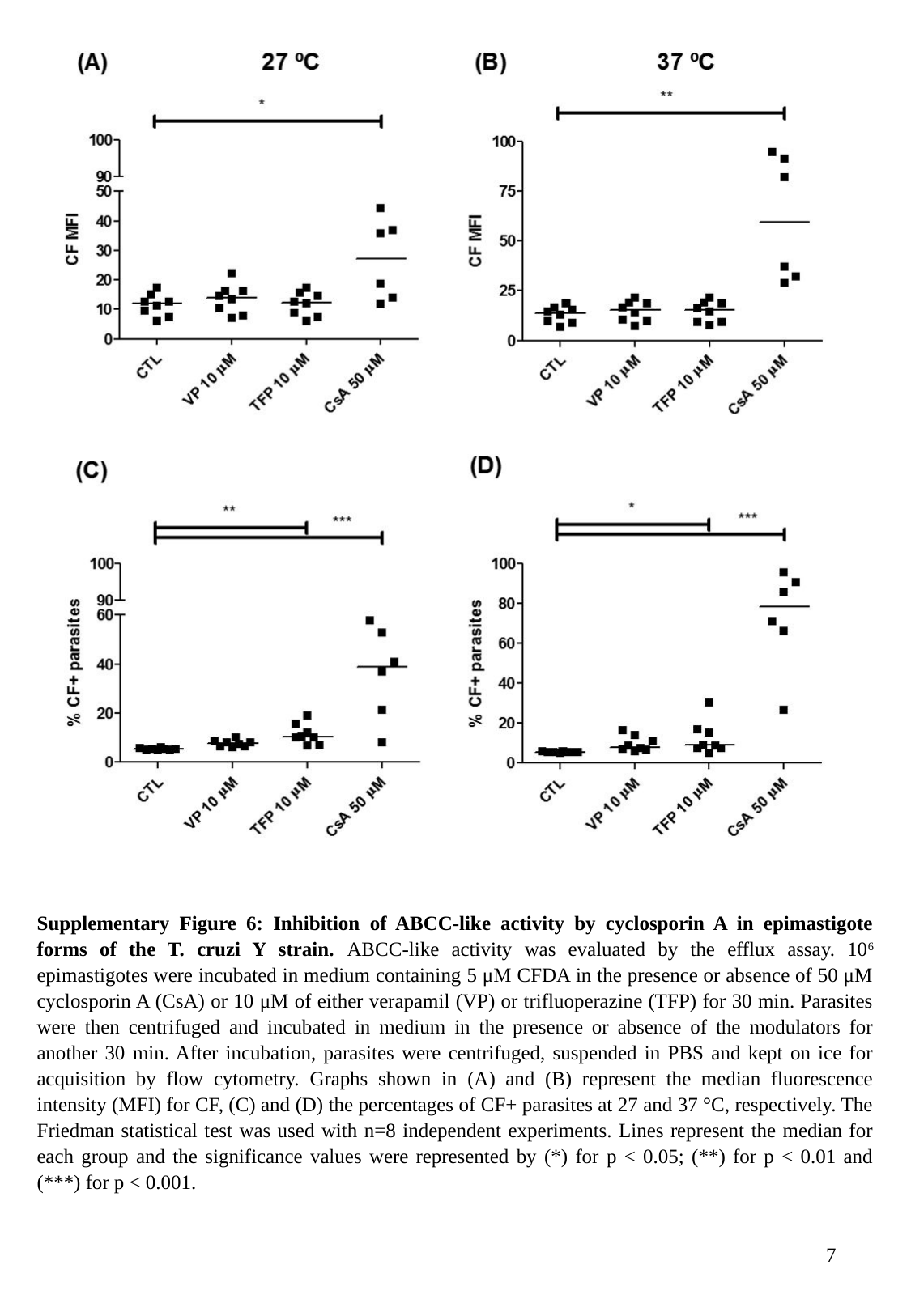

Supplementary Figure 6: Inhibition of ABCC-like activity by cyclosporin A in epimastigote forms of the T. cruzi Y strain. ABCC-like activity was evaluated by the efflux assay. 106 epimastigotes were incubated in medium containing 5 μM CFDA in the presence or absence of 50 μM cyclosporin A (CsA) or 10 μM of either verapamil (VP) or trifluoperazine (TFP) for 30 min. Parasites were then centrifuged and incubated in medium in the presence or absence of the modulators for another 30 min. After incubation, parasites were centrifuged, suspended in PBS and kept on ice for acquisition by flow cytometry. Graphs shown in (A) and (B) represent the median fluorescence intensity (MFI) for CF, (C) and (D) the percentages of CF+ parasites at 27 and 37 °C, respectively. The Friedman statistical test was used with n=8 independent experiments. Lines represent the median for each group and the significance values were represented by (*) for p < 0.05; (**) for p < 0.01 and (***) for p < 0.001.
7

## Slide 8
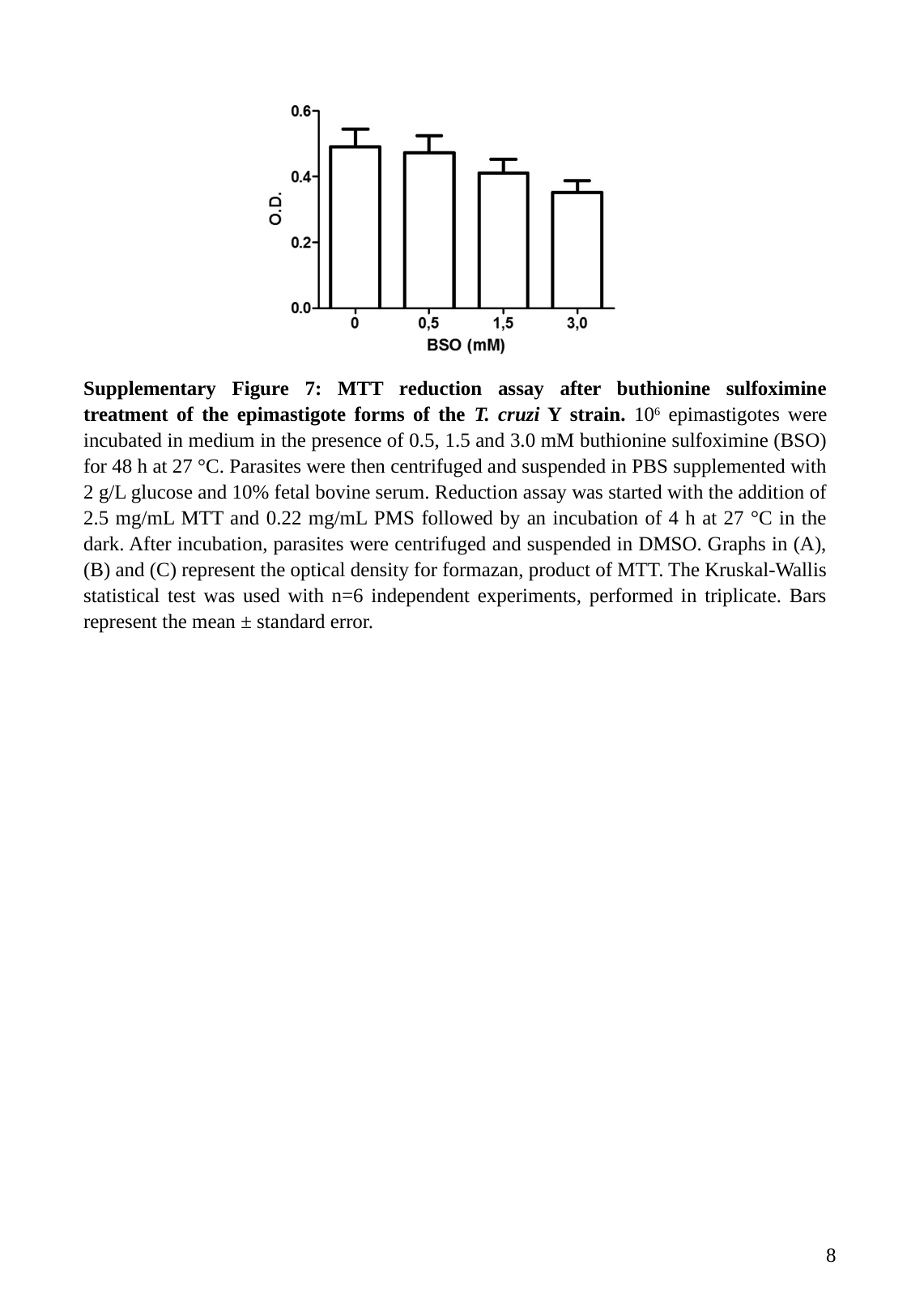

Supplementary Figure 7: MTT reduction assay after buthionine sulfoximine treatment of the epimastigote forms of the T. cruzi Y strain. 106 epimastigotes were incubated in medium in the presence of 0.5, 1.5 and 3.0 mM buthionine sulfoximine (BSO) for 48 h at 27 °C. Parasites were then centrifuged and suspended in PBS supplemented with 2 g/L glucose and 10% fetal bovine serum. Reduction assay was started with the addition of 2.5 mg/mL MTT and 0.22 mg/mL PMS followed by an incubation of 4 h at 27 °C in the dark. After incubation, parasites were centrifuged and suspended in DMSO. Graphs in (A), (B) and (C) represent the optical density for formazan, product of MTT. The Kruskal-Wallis statistical test was used with n=6 independent experiments, performed in triplicate. Bars represent the mean ± standard error.
8

## Slide 9
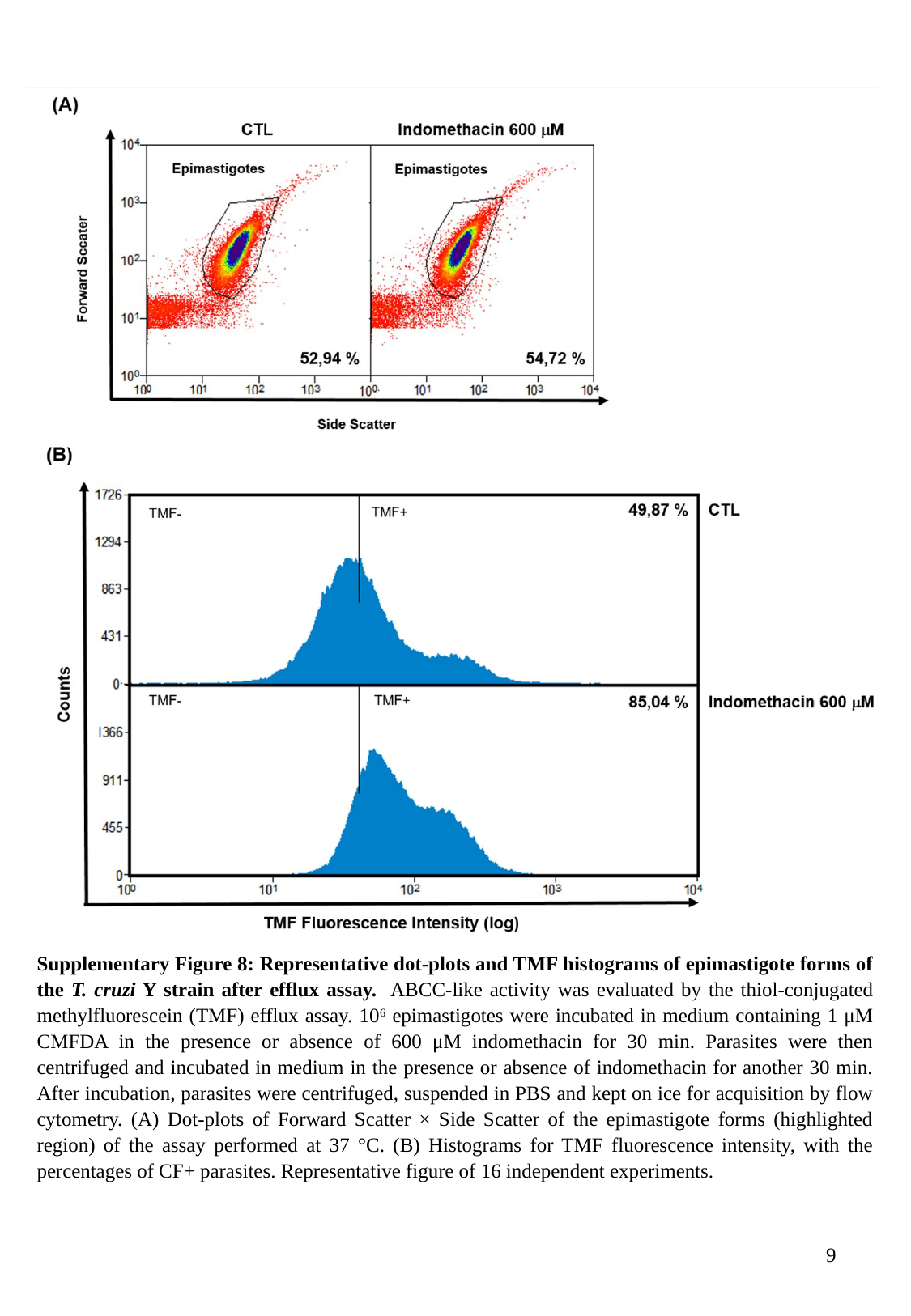

Supplementary Figure 8: Representative dot-plots and TMF histograms of epimastigote forms of the T. cruzi Y strain after efflux assay. ABCC-like activity was evaluated by the thiol-conjugated methylfluorescein (TMF) efflux assay. 106 epimastigotes were incubated in medium containing 1 μM CMFDA in the presence or absence of 600 μM indomethacin for 30 min. Parasites were then centrifuged and incubated in medium in the presence or absence of indomethacin for another 30 min. After incubation, parasites were centrifuged, suspended in PBS and kept on ice for acquisition by flow cytometry. (A) Dot-plots of Forward Scatter × Side Scatter of the epimastigote forms (highlighted region) of the assay performed at 37 °C. (B) Histograms for TMF fluorescence intensity, with the percentages of CF+ parasites. Representative figure of 16 independent experiments.
9

## Slide 10
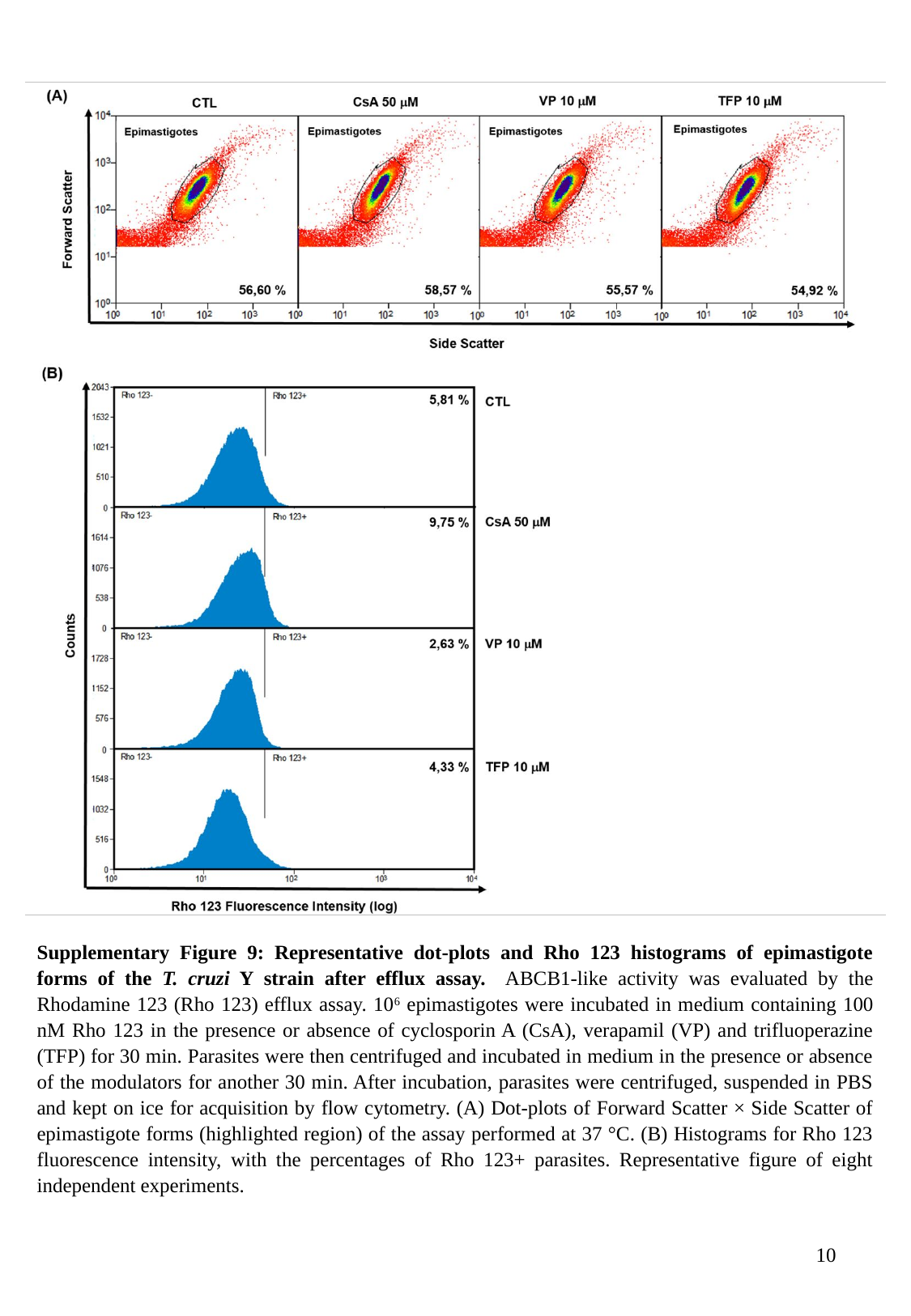

Supplementary Figure 9: Representative dot-plots and Rho 123 histograms of epimastigote forms of the T. cruzi Y strain after efflux assay. ABCB1-like activity was evaluated by the Rhodamine 123 (Rho 123) efflux assay. 106 epimastigotes were incubated in medium containing 100 nM Rho 123 in the presence or absence of cyclosporin A (CsA), verapamil (VP) and trifluoperazine (TFP) for 30 min. Parasites were then centrifuged and incubated in medium in the presence or absence of the modulators for another 30 min. After incubation, parasites were centrifuged, suspended in PBS and kept on ice for acquisition by flow cytometry. (A) Dot-plots of Forward Scatter × Side Scatter of epimastigote forms (highlighted region) of the assay performed at 37 °C. (B) Histograms for Rho 123 fluorescence intensity, with the percentages of Rho 123+ parasites. Representative figure of eight independent experiments.
10

## Slide 11
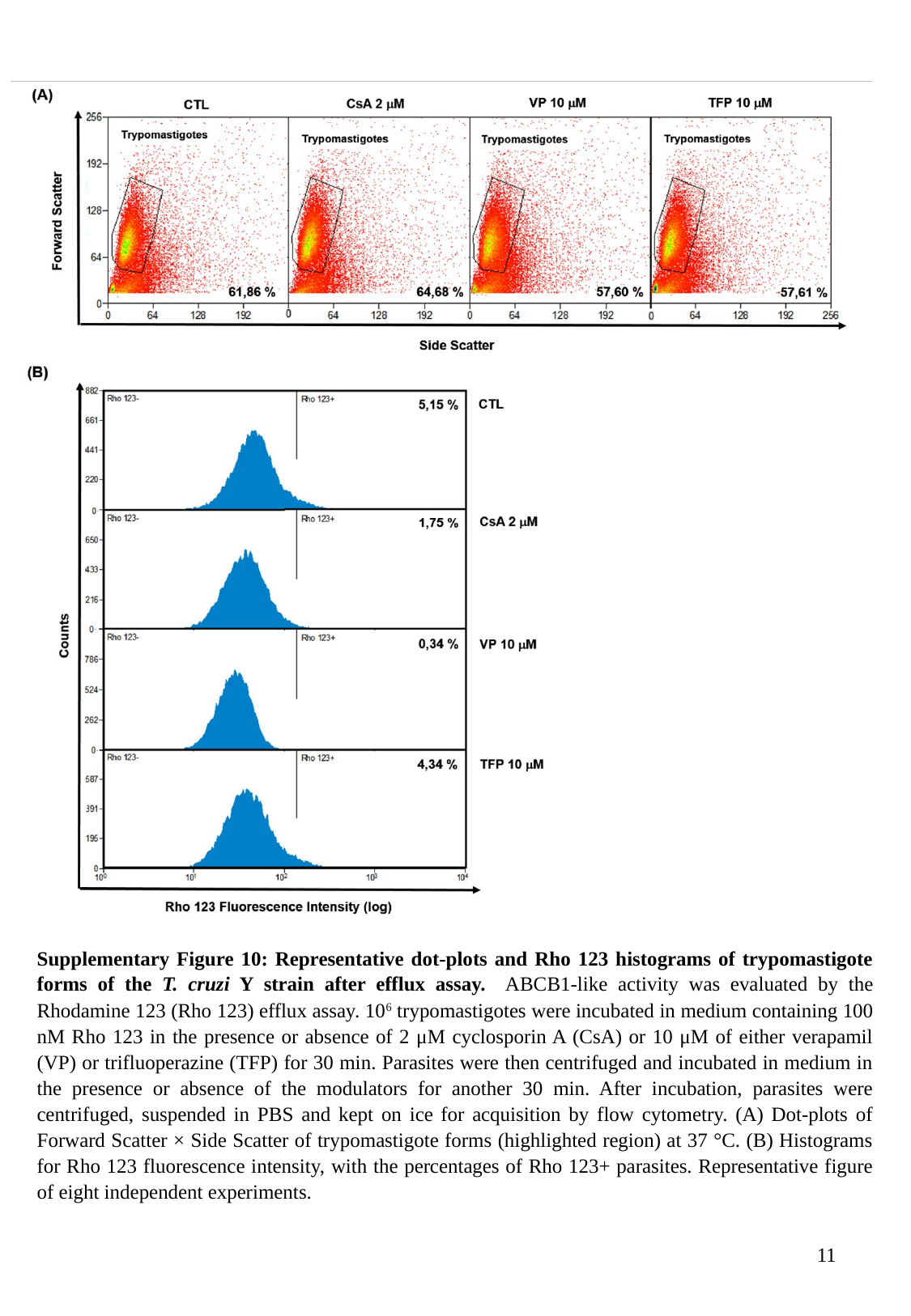

Supplementary Figure 10: Representative dot-plots and Rho 123 histograms of trypomastigote forms of the T. cruzi Y strain after efflux assay. ABCB1-like activity was evaluated by the Rhodamine 123 (Rho 123) efflux assay. 106 trypomastigotes were incubated in medium containing 100 nM Rho 123 in the presence or absence of 2 μM cyclosporin A (CsA) or 10 μM of either verapamil (VP) or trifluoperazine (TFP) for 30 min. Parasites were then centrifuged and incubated in medium in the presence or absence of the modulators for another 30 min. After incubation, parasites were centrifuged, suspended in PBS and kept on ice for acquisition by flow cytometry. (A) Dot-plots of Forward Scatter × Side Scatter of trypomastigote forms (highlighted region) at 37 °C. (B) Histograms for Rho 123 fluorescence intensity, with the percentages of Rho 123+ parasites. Representative figure of eight independent experiments.
11
